# HMGB2 regulates the differentiation and stemness of exhausted CD8^+^ T cells during chronic viral infection and cancer

**DOI:** 10.1038/s41467-023-41352-0

**Published:** 2023-09-13

**Authors:** Emily N. Neubert, Julia M. DeRogatis, Sloan A. Lewis, Karla M. Viramontes, Pedro Ortega, Monique L. Henriquez, Rémi Buisson, Ilhem Messaoudi, Roberto Tinoco

**Affiliations:** 1 Department of Molecular Biology and Biochemistry, School of Biological Sciences, University of California Irvine, Irvine, CA 92697 USA; 2 Center for Virus Research, University of California Irvine, Irvine, CA 92697 USA; 3 La Jolla Institute for Immunology, La Jolla, CA 92037 USA; 4 Department of Biological Chemistry, School of Medicine, University of California, Irvine, Irvine, CA 92697 USA; 5 Center for Epigenetics and Metabolism, Chao Family Comprehensive Cancer Center, University of California Irvine, Irvine, CA 92697 USA; 6 Microbiology, Immunology, and Molecular Genetics, University of Kentucky, Lexington, KY 40536 USA; 7 Institute for Immunology, University of California, Irvine, Irvine, CA 92697 USA; 8 Chao Family Comprehensive Cancer Center, University of California, Irvine, Irvine, CA 92697 USA

**Keywords:** Infection, Cellular immunity, Viral infection

## Abstract

Chronic infections and cancers evade the host immune system through mechanisms that induce T cell exhaustion. The heterogeneity within the exhausted CD8^+^ T cell pool has revealed the importance of stem-like progenitor (Tpex) and terminal (Tex) exhausted T cells, although the mechanisms underlying their development are not fully known. Here we report High Mobility Group Box 2 (HMGB2) protein expression is upregulated and sustained in exhausted CD8^+^ T cells, and HMGB2 expression is critical for their differentiation. Through epigenetic and transcriptional programming, we identify HMGB2 as a cell-intrinsic regulator of the differentiation and maintenance of Tpex cells during chronic viral infection and in tumors. Despite *Hmgb2*^*−/−*^ CD8^+^ T cells expressing TCF-1 and TOX, these master regulators were unable to sustain Tpex differentiation and long-term survival during persistent antigen. Furthermore, HMGB2 also had a cell-intrinsic function in the differentiation and function of memory CD8^+^ T cells after acute viral infection. Our findings show that HMGB2 is a key regulator of CD8^+^ T cells and may be an important molecular target for future T cell-based immunotherapies.

## Abstract (web summary)

T cells can become exhausted during chronic virus infection or repeated stimulation by tumour antigens. Here the authors show that High-Mobility Group Box 2 (HMGB2) protein promotes CD8^+^ T cell memory cell generation in acute viral infection and exhaustion stemness in chronic infection.

## Introduction

During chronic viral infections, there is a dynamic interplay between host and pathogen, where multiple cellular and molecular mechanisms inhibit the immune response and facilitate viral persistence. A key mechanism is the differentiation of exhausted T cells, which are dysfunctional and fail to clear the virus. Despite being less functional than effector CD8^+^ T cells, exhausted T cells still provide some protection to the host^1,2^, which is highlighted in simian immunodeficiency virus studies showing host progression to AIDS-like disease and death when T cells are depleted^3^. Chronic antigen stimulation results in responding T cell dysfunction and heterogeneity, with altered transcription, epigenome, and metabolism unique to exhausted T cells^4–8^. Two key exhausted T cell subsets, defined by phenotype and transcription factor expression, are progenitor exhausted (Tpex) and terminal exhausted (Tex) T cells^9–11^. Tpex cells are long-lived, self-renew, and give rise to Tex cells^11^. They also express the key transcription factors TCF-1, BCL-6, and BACH2^12^. In contrast, Tex cells express high TOX, BLIMP-1, and TIM-3, have increased effector functions, and undergo higher rates of apoptosis^12^. Despite the detailed characterization of these two main subsets, their differentiation mechanisms have not been fully described.

Understanding exhausted T cell heterogeneity has important clinical implications for immune checkpoint blockade (ICB) therapy against chronic viral infections and cancer^13^. Studies have shown anti-PD-1/anti-PD-L1 blockade reinvigorates the Tpex population, which proliferates and further increases numbers of the more cytotoxic Tex cells^14^. Emerging evidence also suggests Tpex cell frequencies may predict patient responsiveness to ICB therapy^9,10,15^ and ability to control HIV viremia^16^. Importantly, exhausted T cells undergo unique epigenetic changes during differentiation, including permanent marks which sustain their exhausted state^17^. Therefore, although exhausted T cells can be re-invigorated with ICB therapy, they revert back to their exhausted phenotype and thereby provide only temporary clinical response in some patients^18–23^. Identifying mechanisms of exhausted T cell differentiation and the associated epigenetic changes remains of high clinical interest as these cells may need to be reprogrammed transcriptionally and/or epigenetically to improve immunotherapy efficacy.

HMGB2 is a member of the High-Mobility Group Box (HMGB) family, which are relatively abundant and highly conserved DNA-binding proteins that modify chromatin structure and regulate gene transcription and transcription factor binding^24–26^. HMGB2 has known roles in regulating stem cells during various differentiation programs, including myogenesis, spermatogenesis, and neurogenesis^27–29^. In mice, *Hmgb2* is expressed early in embryogenesis, but is limited to lymphoid organs and testes in adults^26^. Despite its characterization in numerous cell types, the role of HMGB2 in CD8^+^ T cells has not been investigated. Previous RNA-sequencing analyses found increased *Hmgb2* expression in mouse exhausted CD8^+^ T cells during lymphocytic choriomeningitis virus (LCMV) infection^30^ and increased *HMGB2* expression in CD8^+^ T cells from cancer patients^31–34^. Given HMGB2’s role in both modulating chromatin architecture and regulating stem cells, along with its high gene expression in CD8^+^ T cells, we investigated the function of HMGB2 in effector, memory, and exhausted CD8^+^ T cells.

Here, we show a cell-intrinsic role for HMGB2 in the differentiation and stemness of memory and exhausted CD8^+^ T cells. Effector, memory, and exhausted CD8^+^ T cells have high HMGB2 expression that is sustained with persistent antigen. After acute viral infection, we observe a decrease of *Hmgb2*^*−/−*^ CD8^+^ memory T cells, with defective central memory T cell (Tcm) formation and recall capacity. In response to chronic viral infection, *Hmgb2*^*−/−*^ CD8^+^ T cells show decreased Tpex differentiation. Even though *Hmgb2*^*−/−*^ CD8^+^ T cells express both TCF-1 and TOX, these transcription factors are unable to support the differentiation and maintenance of Tpex and Tex cells. Mechanistically, HMGB2 regulates Tpex-specific transcriptional programming through increasing chromatin accessibility of Tpex genes, while decreasing accessibility of regions specific for Tex cells during chronic infection. Our findings show a role for HMGB2 as an essential regulator of memory and exhausted CD8^+^ T cell differentiation, that protects these cells from a terminal fate.

## Results

### HMGB2 expression is upregulated and sustained in virus-specific CD8^+^ T cells

**Fig. 1:**
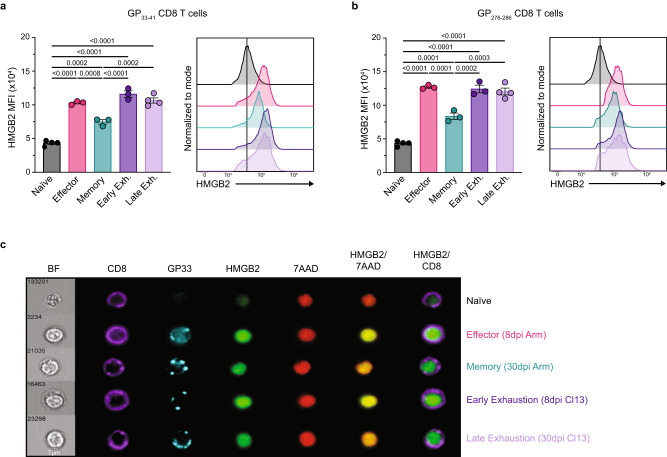
HMGB2 expression in mouse virus-specific CD8^+^ T cells. Expression levels of HMGB2 in splenic GP_33-41_ CD8^+^ T cells (**a**) and GP_276-286_ CD8^+^ T cells (**b**) assessed by flow cytometry; *n* = 3. Naïve, uninfected; Effector, 8dpi LCMV Arm; Memory, 30dpi LCMV Arm; Early Exhaustion, 8dpi LCMV Cl13; Late Exhaustion, 30dpi LCMV Cl13; MFI, mean fluorescence intensity. **c** Representative Imagestream analysis of GP_33-41_ CD8^+^ T cells, magnification, 60x. Data is mean ± s.e.m and representative of two independent experiments. Statistical significance was calculated using a one-way ANOVA followed by Turkey’s multiple comparisons test (**a**, **b**). Source data are provided as a Source Data file.

To assess how HMGB2 is regulated in virus-specific CD8^+^ T cells, we evaluated its expression in naïve, effector, memory, and early and late exhausted T cells. We infected wild-type (WT) mice with either LCMV Armstrong (Arm) or Clone 13 (Cl13) to induce an acute or chronic viral infection, respectively, and measured HMGB2 levels in MHC class I tetramer^+^ virus-specific CD8^+^ T cells. We detected HMGB2 expression in naïve CD8^+^ T cells that was upregulated in GP_33-41_ CD8^+^ effector and memory T cells (Fig. 1a). We observed upregulation of HMGB2 in early exhausted T cells (8dpi Cl13) that was sustained in late exhausted T cells (30dpi Cl13) (Fig. 1a). Furthermore, late exhausted GP_33-41_ T cells had significantly increased HMGB2 expression compared to naïve and memory T cells (Fig. 1a). We next evaluated HMGB2 levels in GP_276-286_ CD8^+^ T cells, and again observed increased levels in effector, memory, and exhausted T cells compared to naïve, with sustained HMGB2 expression in late exhausted CD8^+^ T cells (Fig. 1b). Lastly, we evaluated HMGB2 localization in GP_33-41_ CD8^+^ T cells using imaging flow cytometry. We observed nuclear localization of HMGB2, as shown by HMGB2 and nuclear 7-AAD colocalization staining (Fig. 1c). These findings showed nuclear localization of HMGB2 in virus-specific CD8^+^ T cells, with upregulated and sustained expression in memory and exhausted CD8^+^ T cells during viral infection.

### *Hmgb2*^*−/−*^ CD8^+^ T cells differentiate into effector and memory T cells during acute viral infection

**Fig. 2:**
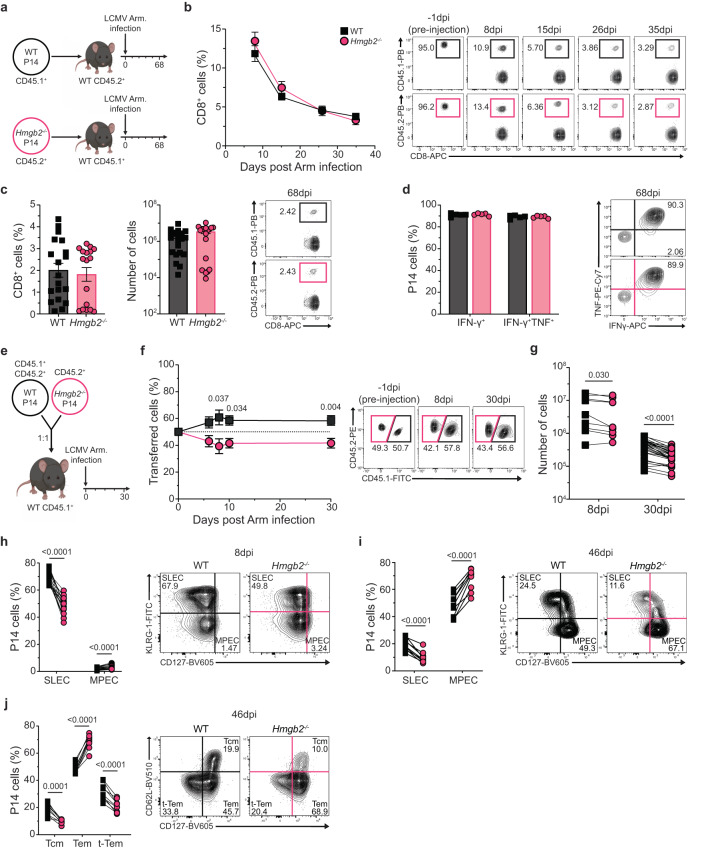
Cell-intrinsic kinetics of WT and *Hmgb2*^*−/−*^ P14 T cells during Arm infection. **a** Experimental scheme for (**b**–**d**). WT and *Hmgb2*^*−/−*^ P14 CD8^+^ T cells were transferred separately into naïve mice and infected with LCMV Arm. Blood taken at 8, 15, 26, and 35dpi. Spleens isolated at 68dpi. **b** Frequency of WT and *Hmgb2*^*−/−*^ P14 T cells of total CD8^+^ population; *n* = 5. **c** Splenic WT and *Hmgb2*^*−/−*^ P14 T cell frequencies and numbers at 68dpi; *n* = 19. **d** Cytokine production by splenic WT and *Hmgb2*^*−/−*^ P14 T cells at 68dpi; *n* = 5. **e** Experimental scheme for (**f**–**j**). WT and *Hmgb2*^*−/−*^ P14 T cells were co-transferred at 1:1 into WT mice and infected with LCMV Arm. Frequencies (**f**) and numbers (**g**) of splenic WT and *Hmgb2*^*−/−*^ P14 T cells at indicated timepoints post-infection; *n* = 5 (**f**), *n* = 9 (**g**, 8dpi), *n* = 28 (**g**, 30dpi). Frequencies of splenic WT and *Hmgb2*^*−/−*^ P14 short-lived effector (SLEC) and memory precursor effector (MPEC) T cells in the blood at 8dpi (**h**) and 46dpi (**i**) Arm; *n* = 20 (**h**), *n* = 7 (**i**). **j** Frequencies of WT and *Hmgb2*^*−/−*^ P14 central memory (Tcm), effector memory (Tem) and terminal Tem (t-Tem) T cells at 46dpi Arm in the spleen; *n* = 9. Data is mean ± s.e.m. Data are representative of three independent experiments except (**c**, **g**, h, **j**) which are cumulative data from three independent experiments. Statistical significance was calculated using an unpaired two-tailed Student’s *t* test followed by Mann–Whitney test (**b**–**d**) or paired two-tailed Student’s *t* test (**f**–**j**). Source data are provided as a Source Data file. (**a**) and (**e**) created with biorender.com.

Considering the expression of HMGB2 was increased in effector and memory CD8^+^ T cells, we next determined the cell-intrinsic role of HMGB2 in virus-specific T cells during acute LCMV infection. Small numbers (1 × 10^3^ cells) of congenically marked WT or *Hmgb2*^*−/−*^ P14 CD8^+^ T cells, which are TCR transgenic T cells specific for the GP_33–41_ peptide of LCMV, were adoptively transferred into separate congenically mismatched WT mice, which were then infected with LCMV Arm (Fig. 2a). A gating strategy for this approach is shown (Supplementary Fig. 1a). We observed similar expansion, contraction, and memory CD8^+^ T cell formation of both WT and *Hmgb2*^*−/−*^ P14 T cells throughout acute infection (Fig. 2b). Furthermore, similar frequencies and numbers of WT and *Hmgb2*^*−/−*^ P14 T cells were found in spleens at 68dpi (Fig. 2c). We next evaluated functionality of WT and *Hmgb2*^*−/−*^ P14 T cells by ex vivo GP_33-41_ peptide stimulation and observed similar frequencies of IFN-γ^+^ and IFN-γ^+^TNF^+^ cells at 68dpi and GranzymeB^+^ cells at 8dpi (Fig. 2d, Supplementary Fig. 2a). To investigate T cell responses within the same host, we next co-transferred small numbers (1×10^3^ each) of congenically marked WT and *Hmgb2*^*−/−*^ P14 T cells at a 1:1 ratio into congenically mismatched WT mice, followed by Arm infection (Fig. 2e). Despite injection at a 1:1 ratio, we observed slightly decreased frequencies of *Hmgb2*^*−/−*^ P14 T cells compared to WT (Fig. 2f). We also observed significantly decreased numbers of splenic *Hmgb2*^*−/−*^ P14 T cells compared to WT at both 8 and 30dpi (Fig. 2g). These findings showed *Hmgb2*^*−/−*^ CD8^+^ T cells expand after acute LCMV infection, and although present at slightly lower frequencies than WT, survive to seed the memory T cell pool.

### HMGB2 regulates memory CD8^+^ T cell differentiation after acute infection

We next evaluated the differentiation of *Hmgb2*^*−/−*^ P14 T cells by examining KLRG-1 and IL-7Rα expression to delineate KLRG1^hi^IL-7Rα^lo^ short-lived effector (SLEC) and KLRG1^lo^IL-7Rα^hi^ memory precursor effector cells (MPEC). At 8dpi, we observed decreased SLECs in *Hmgb2*^*−/−*^ P14 T cells (Fig. 2h) and by 46dpi, we found significantly decreased frequencies of SLECs and increased frequencies of MPECs in *Hmgb2*^*−/−*^ P14 T cells (Fig. 2i). Since we found more *Hmgb2*^*−/−*^ MPECs compared to WT but significantly less overall *Hmgb2*^*−/−*^ memory T cell numbers (Fig. 2g), we investigated apoptosis within this population. We found *Hmgb2*^*−/−*^ MPECs had significantly higher frequencies of total apoptotic cells by active Caspase3 and propidium iodide (PI) staining at 46dpi (Supplementary Fig. 2b).

To further characterize the differentiation of memory *Hmgb2*^*−/−*^ P14 T cells, we examined effector memory (Tem), central memory (Tcm) and terminal-Tem (t-Tem) T cell populations based on CD62L vs CD127 expression^35^. *Hmgb2*^*−/−*^ P14 T cells had significantly lower frequencies of both Tcm and t-Tem cells, and increased frequencies of Tem cells compared to WT at 46dpi (Fig. 2j). Finally, to confirm the loss of *Hmgb2*^*−/−*^ P14 T cells was not due to compromised development of naïve CD8^+^ T cells, we characterized naïve WT and *Hmgb2*^*−/−*^ P14 T cells from spleens and lymph nodes of uninfected mice. We found no differences in expression of naïve T cell markers (CD127, CD62L, CCR7) or changes in frequencies of naïve vs activated T cells in lymph nodes (Supplementary Fig. 3a, b) and spleens (Supplementary Fig. 3c, d). We also found no differences in numbers of CD8^+^ T cells between WT and *Hmgb2*^*−/−*^ mice (Supplementary Fig. 3e). Together, these findings showed that HMGB2 regulated the survival and differentiation of memory CD8^+^ T cells during acute viral infection.

### Cell-intrinsic HMGB2 expression is essential for long-term maintenance of exhausted CD8^+^ T cells

**Fig. 3:**
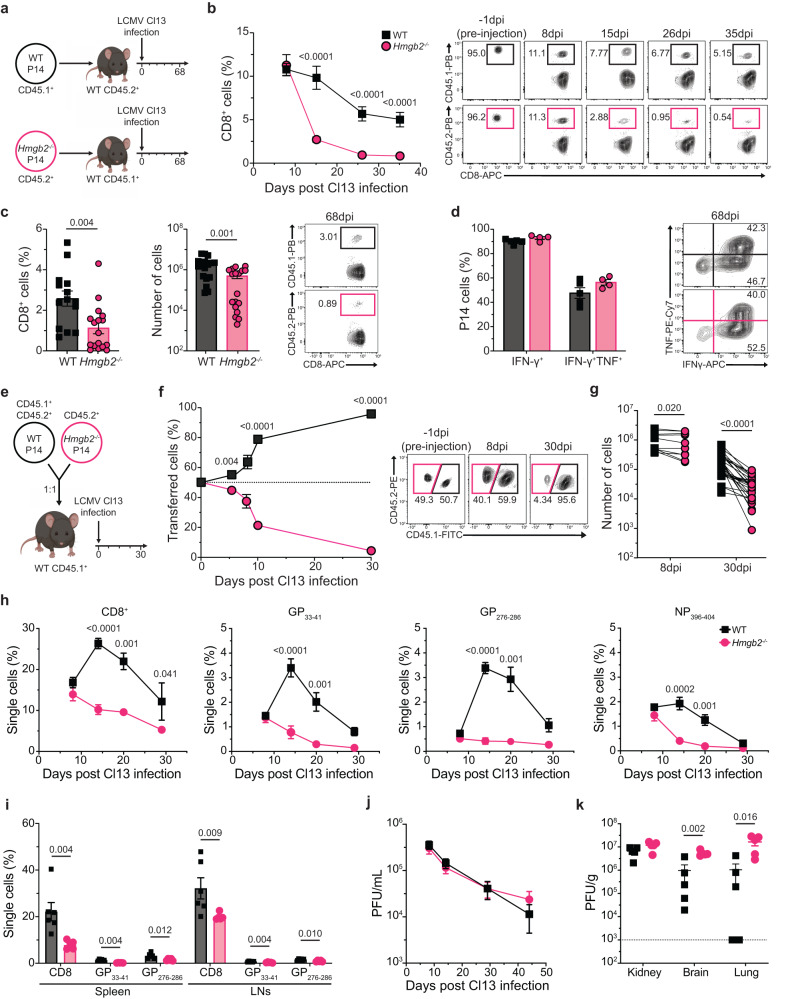
Cell-intrinsic kinetics of WT and *Hmgb2*^*−/−*^ P14 T cells during Cl13 infection. **a** Experimental scheme for (**b**–**d**). WT and *Hmgb2*^*−/−*^ P14 CD8^+^ T cells were transferred separately into naïve mice and infected with LCMV Cl13. Blood taken at 8, 15, 26, and 35dpi. Spleens isolated at 68dpi. **b** Frequency of WT and *Hmgb2*^*−/−*^ P14 T cells of total CD8^+^ population; *n* = 5. **c** Splenic WT and *Hmgb2*^*−/−*^ P14 T cell frequencies and numbers at 68dpi Cl13; *n* = 17. **d** Cytokine production by splenic WT and *Hmgb2*^*−/−*^ P14 T cells at 68dpi Cl13; *n* = 5. **e** Experimental scheme for (**f**–**g**). WT and *Hmgb2*^*−/−*^ P14 T cells were co-transferred at 1:1 into WT mice and infected with Cl13. Frequencies (**f**) and numbers (**g**) of splenic WT and *Hmgb2*^*−/−*^ P14 T cells at indicated timepoints post infection; *n* = 5 (**f**), *n* = 10 (**g**, 8dpi), *n* = 28 (**g**, 30dpi). **h** Frequencies of total CD8^+^, GP_33-41_, GP_276-286_, and NP_396-404_ T cells during Cl13 infection in the blood of WT and *Hmgb2*^*−/−*^ mice; *n* = 9. **i** Frequencies of total CD8^+^, GP_33-41_, GP_276-286_, and NP_396-404_ T cells during Cl13 infection in the spleen and lymph nodes (LNs) of WT and *Hmgb2*^*−/−*^ mice at 44dpi; *n* = 6. **j** Virus titers in serum at 44dpi by plaque forming units (PFU) and expressed as PFU/mL; *n* = 9. **k** Virus titers in the kidney, brain, and lung, expressed as PFU/g; *n* = 5. Limits of detection are indicated by dashed lines. Data is mean ± s.e.m. Data are representative of two or more independent experiments except (**c**, **g**, **h**, **j**) which are cumulative data from two or more independent experiments. Statistical significance was calculated using an unpaired two-tailed Student’s *t* test followed by Mann–Whitney test (**b**–**d**, **h**–**k**) or paired two-tailed Student’s *t* test (**f**–**g**). Source data are provided as a Source Data file. (**a**) and (**e**) created with biorender.com.

Since HMGB2 expression was increased and sustained in exhausted CD8^+^ T cells, we next determined the cell-intrinsic role of HMGB2 in virus-specific T cells during chronic LCMV infection. Small numbers (1 × 10^3^ cells) of congenically marked WT or *Hmgb2*^*−/−*^ P14 T cells were adoptively transferred into congenically mismatched WT mice and infected with LCMV Cl13 (Fig. 3a). We observed similar expansion of both WT and *Hmgb2*^*−/−*^ P14 T cells at 8dpi, but *Hmgb2*^*−/−*^ P14 T cells had an accelerated decline and were present at significantly lower frequencies compared to WT by 15dpi (Fig. 3b). Furthermore, we observed significantly decreased frequencies and numbers of *Hmgb2*^*−/−*^ P14 T cells in spleens at 68dpi (Fig. 3c). We next evaluated the functionality of WT and *Hmgb2*^*−/−*^ P14 T cells and observed similar frequencies of IFN-γ^+^ and IFN-γ^+^TNF^+^ T cells at 68dpi and GranzymeB^+^ cells at 8dpi (Fig. 3d, S4a). To investigate cell-intrinsic HMGB2 function during chronic infection, we co-transferred WT and *Hmgb2*^*−/−*^ P14 T cells at a 1:1 ratio into WT mice and then infected with Cl13 (Fig. 3e). Despite starting at a 1:1 ratio, we observed a significant decrease in frequencies of *Hmgb2*^*−/−*^ P14 T cells compared to WT cells as early as 8dpi (Fig. 3f). Additionally, we observed significantly decreased numbers of *Hmgb2*^*−/−*^ P14 T cells compared to WT at 8 and 30dpi (Fig. 3g). We next evaluated co-inhibitory receptor expression and found that *Hmgb2*^*−/−*^ CD8^+^ T cells were increased in PD-1^+^TIM-3^+^ cells throughout Cl13 infection (Supplementary Fig. 4b). Furthermore, we observed higher PD-1 expression in *Hmgb2*^*−/−*^ P14 T cells at 26 and 35dpi (Supplementary Fig. 4c). Since it has been established TOX is required for the generation of exhausted T cells during Cl13 infection^36–38^, we next asked if the loss of exhausted *Hmgb2*^*−/−*^ P14 T cells was due to decreased TOX expression compared to WT. Significantly, we found *Hmgb2*^*−/−*^ P14 T cells had both similar and increased frequencies of TOX^+^ cells compared to WT, but had impaired generation of exhausted T cells (Supplementary Fig. 4d).

Since we saw diminished maintenance of exhausted *Hmgb2*^*−/−*^ P14 T cells, we wanted to investigate the impact of HMGB2 deletion on other virus-specific T cell clones by infecting WT and *Hmgb2*^*−/−*^ mice with Cl13. By 8dpi there was similar expansion of CD8^+^, GP_33-41_, GP_276-286_, and NP_396-404_ T cells in both WT and *Hmgb2*^*−/−*^ mice, but by 14dpi and onwards there were significantly less virus-specific CD8^+^ T cells in *Hmgb2*^*−/−*^ mice (Fig. 3h). At 44dpi, there were significantly decreased frequencies of CD8^+^, GP_33-41_, and GP_276-286_ T cells in spleens and lymph nodes of *Hmgb2*^*−/−*^ mice compared to WT (Fig. 3i). Throughout the course of infection, there were no differences in serum viral titers but we observed significantly increased viral titers in the brains and lungs of *Hmgb2*^*−/−*^ mice at 44dpi (Fig. 3j, k). Together, these findings showed HMGB2 was essential for the formation and long-term maintenance of exhausted CD8^+^ T cells during chronic viral infection.

### HMGB2 regulates the transcriptional signature of progenitor-exhausted CD8^+^ T cells

**Fig. 4:**
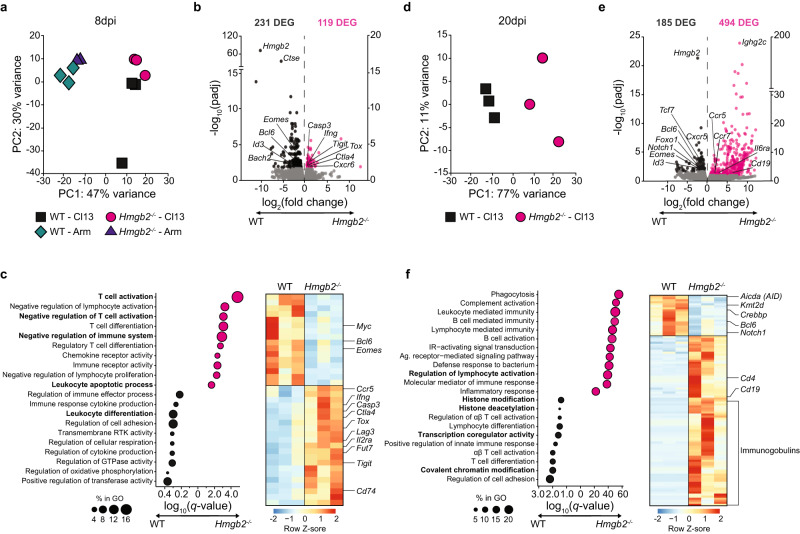
*Hmgb2*^*−/−*^ CD8^+^ T cells have decreased expression of Tpex signature genes. Bulk RNA-seq analysis of WT and *Hmgb2*^*−/−*^ P14 T cells during LCMV Arm and Cl13 infection. **a** Principal component analysis (PCA) of WT and *Hmgb2*^*−/−*^ P14 T cells at 8 days post either Arm or Cl13 infection. **b** Volcano plot highlighting differentially expressed genes (DEG) between WT and *Hmgb2*^*−/−*^ P14 T cells at 8dpi Cl13. Significant DEG (padj ≤ 0.1, |log_2_FC | ≥ 0.5) were determined using *DESeq2* and are colored (pink = upregulated in *Hmgb2*^*−/−*^ P14 T cells; black = upregulated in WT P14 T cells). **c** Left: Gene ontology (GO) biological process enrichment from Metascape of significant DEG from (**b**). *X*-axis represents log_10_(*q*-value) and size of dot represents proportion of the total DEG enriched to that given pathway. Right: Heatmap of average normalized expression of genes associated with bolded pathways. Each column represents one independent experiment with *n* = 5 mice pooled. **d** PCA of co-transferred WT and *Hmgb2*^*−/−*^ P14 T cells at 20dpi Cl13. **e** Volcano plot highlighting DEG between WT and *Hmgb2*^*−/−*^ P14 T cells at 20dpi Cl13. Significant DEG (padj ≤ 0.1, |log_2_FC | ≥ 0.5) were determined using *DESeq2* and are colored (pink = upregulated in *Hmgb2*^*−/−*^ P14 T cells; black = upregulated in WT P14 T cells). **f** Left: GO biological process enrichment from Metascape of DEG from (**e**). *X*-axis represents log_10_(*q*-value) and size of dot represents proportion of the total DEG enriched to that given pathway. Right: Heatmap of average normalized expression of genes associated with bolded pathways. Each column represents one independent experiment with *n* = 10 mice pooled.

Since we found roles for HMGB2 in memory and exhausted CD8^+^ T cells, and HMGB2 is a chromatin modifier, we next investigated whether HMGB2 regulated the transcriptional landscape of virus-specific T cells. We performed RNA-sequencing of sorted WT and *Hmgb2*^*−/−*^ P14 T cells from Arm and Cl13 infected mice at 8dpi. Principal component analysis (PCA) showed the type of infection accounted for transcriptional differences across PC1 (47% variance), while *Hmgb2* expression accounted for transcriptional changes across PC2 (30% variance) (Fig. 4a). Overall, we observed fewer differentially expressed genes (DEG) between WT and *Hmgb2*^*−/−*^ P14 T cells during Arm infection than Cl13. During Arm infection, analysis of DEG between WT and *Hmgb2*^*−/−*^ P14 T cells showed a total of 125, with 38 upregulated and 87 downregulated genes in *Hmgb2*^*−/−*^ P14 T cells (Supplementary Fig. 5a, b). Conversely, there were 350 DEG with 119 upregulated and 231 downregulated genes in *Hmgb2*^*−/−*^ P14 T cells during Cl13 infection (Fig. 4b). In WT P14 T cells, we observed increased expression of genes promoting stem-like progenitor exhausted T cell (Tpex) differentiation (*Eomes*, *Bcl6*, *Id3*, *Bach2*), whereas *Hmgb2*^*−/−*^ P14 T cells had increased expression of genes associated with terminal exhausted T cells (Tex) (*Casp3*, *Tigit*, *Tox*, *Ctla4*, *Ifng*) (Fig. 4b)^39^. To identify the biological significance of these DEG during Cl13 infection, we performed gene ontology (GO) analysis. We found WT P14 T cells were enriched for pathways associated with (i) leukocyte differentiation; (ii) regulation of cellular respiration and oxidative phosphorylation; and (iii) transferase activity (Fig. 4c). In contrast, *Hmgb2*^*−/−*^ P14 T cells were enriched for pathways associated with (i) negative regulation of T cell activation; (ii) immune receptor activity; and (iii) apoptosis (Fig. 4c). These findings showed *Hmgb2*^*−/−*^ CD8^+^ T cells are enriched for gene signatures and pathways of terminal exhaustion during chronic viral infection.

### HMGB2 is required for expression of Tpex genes, including genes regulated by TCF-1

To further investigate the transcriptional changes driven by HMGB2 in CD8^+^ T cells during chronic infection, we performed RNA-sequencing on WT and *Hmgb2*^*−/−*^ P14 T cells sorted from Cl13 infected mice at 20dpi. PCA showed clear separation of WT and *Hmgb2*^*−/−*^ P14 T cells, with *Hmgb2* expression accounting for the transcriptional differences across PC1 (77% variance) (Fig. 4d). There were 679 DEG, with 494 upregulated and 185 downregulated in *Hmgb2*^*−/−*^ P14 T cells (Fig. 4e). Similar to 8dpi Cl13, we observed increased expression of Tpex associated genes in WT P14 T cells (*Tcf7*, *Cxcr5*, *Bcl6*, *Foxo1*, *Eomes*, *Id3*) compared to *Hmgb2*^*−/−*^ P14 T cells (Fig. 4e)^39^. *Hmgb2*^*−/−*^ P14 T cells expressed a dysregulated gene expression program, including non-CD8^+^ T cell lineage associated genes (*Ighg2c, Cd19*) (Fig. 4e). GO term analysis showed WT P14 T cells were enriched for pathways associated with (i) histone modification; (ii) histone deacetylation; (iii) lymphocyte differentiation; (iv) transcription coregulator activity; and (v) covalent chromatin modification (Fig. 4f). In contrast, *Hmgb2*^*−/−*^ P14 T cells were enriched for pathways associated with (i) phagocytosis; (ii) complement activation; (iii) B cell activation; (iv) regulation of lymphocyte activation; and (v) inflammatory responses (Fig. 4f).

Since HMGB proteins are also known to modulate transcription factor binding, we next used Ingenuity Pathway Analysis (IPA)^40^ to investigate any transcriptional regulators modified by HMGB2 that may be responsible for the DEG between WT and *Hmgb2*^*−/−*^ P14 T cells at 20dpi Cl13. Upstream causal network analysis identified TCF-1 as a possible master regulator of the 20dpi Cl13 DEG, with the TCF-1 regulator network predicated to be significantly inhibited in exhausted *Hmgb2*^*−/−*^ P14 T cells (activation *z*-score = −3.236, network bias-corrected *p*-value = 0.00001) (Supplementary Fig. 6a). Of the 679 DEG regulated by HMGB2 at 20dpi Cl13, 117 are downstream targets of TCF-1, including *Bcl6*, *Eomes*, *Id3*, *Foxo1*, *Notch1*, and *Tcf7* (Supplementary Fig. 6a). This suggests that HMGB2 in CD8^+^ T cells regulates the Tpex transcription program during Cl13 infection, and may do so through modifying the TCF-1 transcriptional network.

### *Hmgb2*^*−/−*^ CD8^+^ T cells have decreased survival during acute and chronic viral infection

**Fig. 5:**
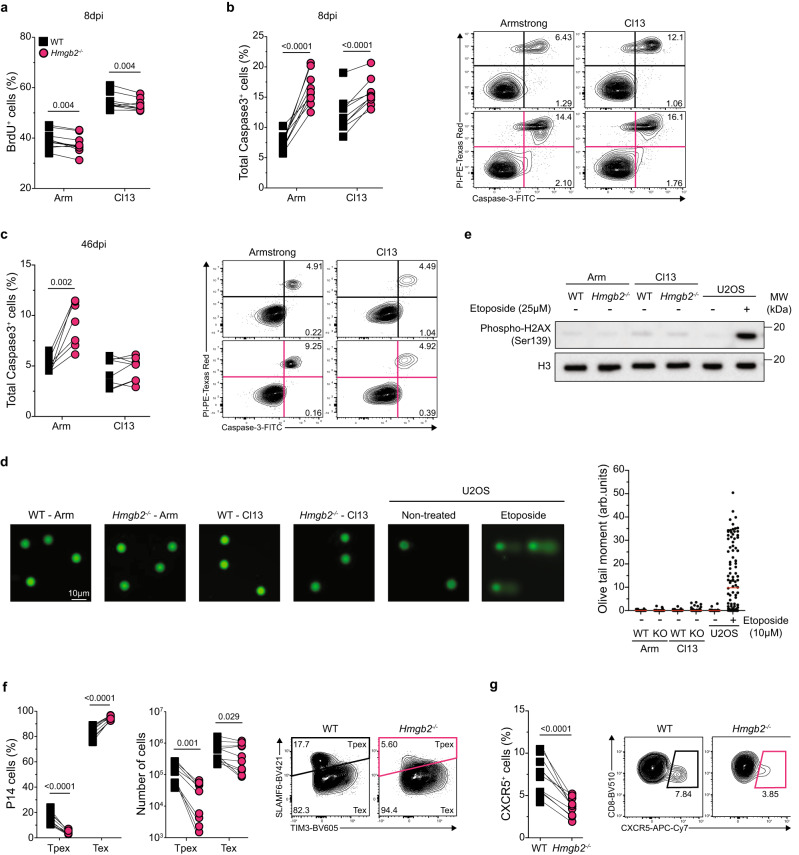
*Hmgb2*^*−/−*^ CD8^+^ T cells have decreased progenitor exhausted T cell differentiation. WT and *Hmgb2*^*−/−*^ P14 T cells were co-transferred at 1:1 into WT mice, followed by LCMV Arm or Cl13 infection. **a** BrdU uptake of splenic WT and *Hmgb2*^*−/−*^ P14 T cells at 8dpi; *n* = 10. Caspase3 and PI staining of P14 T cells at 8dpi (**b**) and 46dpi (**c**) in the spleen; *n* = 10 (**b**), *n* = 8 (**c**). **d** Alkaline comet assay of splenic WT and *Hmgb2*^*−/−*^ P14 T cells isolated on 8 days post either Arm or Cl13 infection (pooled samples from *n* = 10). Representative fluorescent comet images of cells stained with Vista Green DNA dye (100 cells were imaged per condition). U2OS human cells treated with etoposide (10 µM for 30 min), a topoisomerase II inhibitor used to generate DNA double-strand breaks in cells, served as controls for comet tail formation. **e** p-H2AX (Ser139) protein expression by western blot in purified splenic WT and *Hmgb2*^*−/−*^ P14 T cells isolated on 8 days post either Arm or Cl13 infection (pooled samples from *n* = 10). U2OS human cells untreated or treated with 25 µM etoposide for 60 min served as negative and positive controls, respectively. **f** Frequencies and numbers of splenic progenitor exhausted (Tpex) and terminal exhausted (Tex) T cells at 8dpi Cl13; *n* = 9. **g** Frequencies of CXCR5^+^ P14 T cells at 8dpi Cl13 in the spleen; *n* = 10. Data is mean ± s.e.m. Data are representative of three independent experiments except (**d**, **e**). Statistical significance was calculated using a paired two-tailed Student’s *t* test (**a**–**c**, **f**–**g**) or an unpaired two-tailed Student’s *t* test followed by Mann–Whitney test (**d**). Source data are provided as a Source Data file.

Since we found *Hmgb2*^*−/−*^ CD8^+^ T cells were significantly decreased during acute and chronic viral infection, we next assessed whether there were differences in their proliferation and/or survival. We co-transferred WT and *Hmgb2*^*−/−*^ P14 T cells at a 1:1 ratio into WT mice, followed by Arm or Cl13 infection. We first evaluated proliferation by in vivo BrdU incorporation and observed slightly decreased frequencies of BrdU^+^ *Hmgb2*^*−/−*^ P14 T cells compared to WT at both 8dpi Arm and Cl13 (Fig. 5a). As expected, WT and *Hmgb2*^*−/−*^ P14 T cells from chronically infected mice had more proliferation than those from acute infection (Fig. 5a). Next, we evaluated survival by active Caspase3 and PI staining and observed increased frequencies of total Caspase3^+^ apoptotic *Hmgb2*^*−/−*^ P14 T cells compared to WT at 8dpi Arm and Cl13 (Fig. 5b). We also found increased frequencies of total Caspase3^+^ apoptotic *Hmgb2*^*−/−*^ P14 T cells at 46dpi Arm but saw minor differences at 46dpi Cl13 (Fig. 5c). We next asked whether the increased apoptosis of *Hmgb2*^*−/−*^ P14 T cells was due to differences in DNA repair compared to WT cells. Using a comet assay to measure DNA damage^41^, we found no significant differences between WT and *Hmgb2*^*−/−*^ P14 T cells during both Arm and Cl13 infection (Fig. 5d). We also looked at phosphorylated-H2AX (Ser139), a marker of DNA damage^42^, and again found no differences between WT and *Hmgb2*^*−/−*^ P14 T cells during both Arm and Cl13 infection (Fig. 5e). These findings showed *Hmgb2*^*−/−*^ CD8^+^ T cells had decreased proliferation and survival compared to WT during both acute and chronic viral infection and did not show signs of DNA damage by 8dpi.

### HMGB2 is essential for the differentiation of progenitor-exhausted T cells during chronic viral infection

Recent findings have shown heterogeneity within the exhausted CD8^+^ T cell population, including the identification of Tpex and Tex cells. Since our sequencing data showed decreased expression of Tpex signature genes in *Hmgb2*^*−/−*^ P14 T cells compared to WT (*Tcf7, Eomes, Bcl6, Id3*), we wanted to determine changes in Tpex differentiation between WT and *Hmgb2*^*−/−*^ P14 T cells. At 8dpi Cl13, we stained adoptively co-transferred P14 T cells with SLAMF6 and TIM-3 to identify Tpex (SLAMF6^hi^TIM-3^lo^) and Tex (SLAMF6^lo^TIM-3^hi^) cells. We found that *Hmgb2*^*−/−*^ P14 T cells had significantly diminished Tpex cell frequencies compared to WT (Fig. 5f). Consistent with the loss of the Tpex subset, *Hmgb2*^*−/−*^ P14 T cells were enriched for Tex-phenotype cells (Fig. 5f). Furthermore, we observed a significant decrease in the number of *Hmgb2*^*−/−*^ Tpex cells at both 8 and 46dpi, while the numbers of Tex cells were similar (Fig. 5f, Supplementary 7a). We also found significantly decreased CXCR5^+^ *Hmgb2*^*−/−*^ P14 T cells compared to WT, which is an additional surface marker of the Tpex population (Fig. 5g). Since we found decreased *Tcf7* expression in *Hmgb2*^*−/−*^ P14 T cells and TCF-1 (*Tcf7*) is a key transcription factor driving Tpex cell differentiation, we investigated if the loss of *Hmgb2*^*−/−*^ Tpex cells was due to decreased TCF-1 expression compared to WT. Surprisingly, WT and *Hmgb2*^*−/−*^ P14 T cells had similar frequencies of TCF-1^+^ cells during Cl13 infection, although overall frequencies of TCF-1^+^ *Hmgb2*^*−/−*^ P14 T cells were significantly decreased compared to TCF-1^+^ WT P14 T cells (Supplementary Fig. 7b, c). In *Hmgb2*^*−/−*^ mice, we also found similar and even higher frequencies of TCF-1^+^ cells in *Hmgb2*^*−/−*^ CD8^+^, GP_33-41_, GP_276-286_, and NP_396–404_ T cells compared to WT mice during Cl13 infection (Supplementary Fig. 7d). However, the overall frequencies of TCF-1^+^ *Hmgb2*^*−/−*^ antigen-specific CD8^+^ T cells were significantly decreased compared to WT (Supplementary Fig. 7e).

Given the substantial loss of *Hmgb2*^*−/−*^ Tpex cells and the overall increased cell death of exhausted *Hmgb2*^*−/−*^ P14 T cells, we next investigated if *Hmgb2*^*−/−*^ Tpex cells were preferentially dying. At 8dpi Cl13, we found no differences in Tpex cell death between WT and *Hmgb2*^*−/−*^ P14 T cells (Supplementary Fig. 8a) but found a significant increase in apoptosis of *Hmgb2*^*−/−*^ Tpex cells compared to WT at 46dpi (Supplementary Fig. 8b).

Lastly, since we found HMGB2 played a critical role in the formation of Tpex cells, we wanted to evaluate the regulation of HMGB2 in this exhausted subset. Within WT P14 T cells, we found the highest expression of HMGB2 in the Tpex subset compared to Tex (Supplementary Fig. 8c). We also found the highest frequencies of Tpex cells within the WT P14 HMGB2^+^ population compared to the WT P14 HMGB2^−^ population (Supplementary Fig. 8d). Together, these findings showed that HMGB2 is a critical regulator of the differentiation and preservation of Tpex cells, which self-renew and maintain the exhausted T cell pool during Cl13 infection.

### *Hmgb2*^*−/−*^ memory CD8^+^ T cells are defective in their recall to secondary infection

**Fig. 6:**
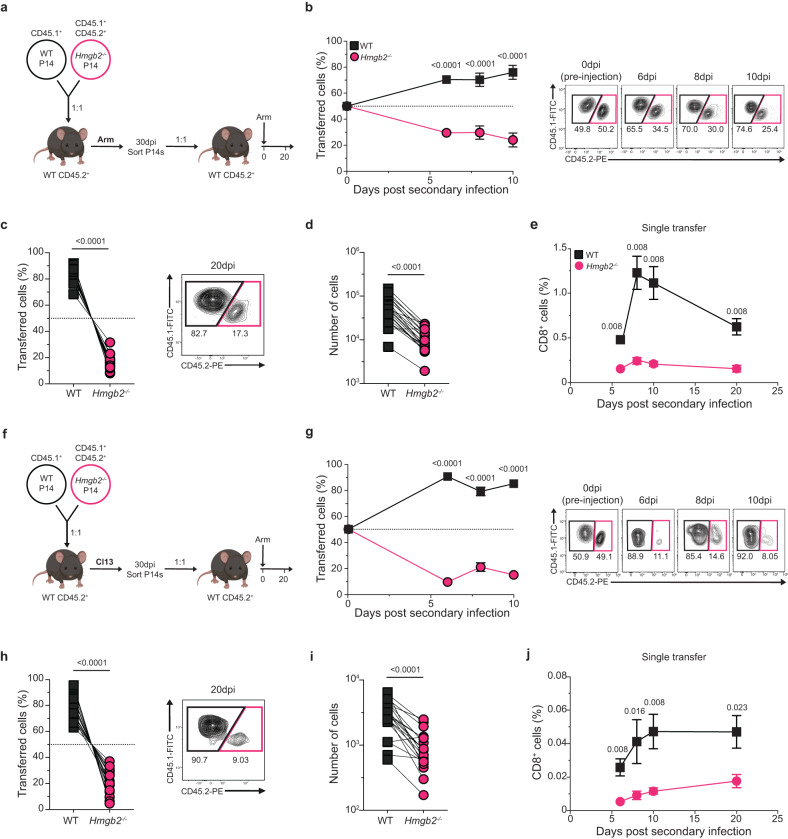
Memory and exhausted *Hmgb2*^*−/−*^ CD8^+^ T cells are defective in their recall capacity. **a** Experimental scheme for (**b**-**d**). WT and *Hmgb2*^*−/−*^ P14 T cells were co-transferred into WT mice at 1:1, followed by LCMV Arm infection. At 30dpi, memory WT and *Hmgb2*^*−/−*^ P14 T cells were sorted and normalized to 1:1 before co-transferred into new naïve mice, followed by Arm infection (secondary infection). **b** Frequency of WT and *Hmgb2*^*−/−*^ P14 T cells in blood during secondary Arm infection; *n* = 8. Frequency (**c**) and number (**d**) of splenic WT and *Hmgb2*^*−/−*^ P14 T cells at 20dpi secondary Arm; *n* = 9 (**c**), *n* = 10 (**d**). **e** WT and *Hmgb2*^*−/−*^ P14 T cells were transferred separately into WT hosts and sorted at 68dpi Arm before adoptive transfer into separate naïve mice, followed by Arm infection (secondary infection). Frequency of WT and *Hmgb2*^*−/−*^ P14 T cells in the blood during secondary Arm infection; *n* = 5. **f** Experimental scheme for (**g**–**i**). WT and *Hmgb2*^*−/−*^ P14 T cells were co-transferred into WT mice at 1:1, followed by LCMV Cl13 infection. At 30dpi, exhausted WT and *Hmgb2*^*−/−*^ P14 T cells were sorted and normalized to 1:1 before co-transferred into new naïve mice, followed by Arm infection (secondary infection). **g** Frequency of WT and *Hmgb2*^*−/−*^ P14 T cells in blood during secondary Arm infection; *n* = 10. Frequency (**h**) and number (**i**) of splenic WT and *Hmgb2*^*−/−*^ P14 T cells at 20dpi secondary Arm; *n* = 9 (**h**), *n* = 10 (**i**). **j** WT and *Hmgb2*^*−/−*^ P14 T cells were transferred separately into WT hosts and sorted at 68dpi Arm before adoptive transfer into separate naïve mice, followed by Arm infection (secondary infection). Frequency of WT and *Hmgb2*^*−/−*^ P14 T cells in the blood during secondary Arm infection; *n* = 5. Data is mean ± s.e.m. Data are representative of three independent experiments. Statistical significance was calculated using a paired two-tailed Student’s *t* test (**b**–**d**, **g**–**i**) or an unpaired two-tailed Student’s *t* test followed by Mann–Whitney test (**e, j**). Source data are provided as a Source Data file.

We found that *Hmgb2*^*−/−*^ P14 T cells survived to form memory T cells, but were deficient in Tcm formation (Fig. 2j). Since Tcm cells can self-renew to maintain the memory T cell pool and mediate memory T cell recall responses^35,43^, we next assessed whether HMGB2 played a role in the functionality of memory CD8^+^ T cells. Small numbers (1 × 10^3^ cells/each) of WT and *Hmgb2*^*−/−*^ P14 T cells were adoptively co-transferred at a 1:1 ratio into WT mice and subsequently infected with LCMV Arm (Fig. 6a). At 30dpi, memory P14 T cells were sorted from spleens and co-transferred (2 × 10^3^ cells/each) at a 1:1 ratio into naïve WT mice, which were then infected with LCMV Arm (Fig. 6a). WT P14 T cells robustly re-expanded with secondary challenge, while *Hmgb2*^*−/−*^ P14 T cell frequencies were significantly decreased in the blood (Fig. 6b). Furthermore, we observed decreased frequencies and numbers of *Hmgb2*^*−/−*^ P14 T cells in spleens at 20dpi (Fig. 6c, d). Similar results were seen when memory WT and *Hmgb2*^*−/−*^ P14 T cells were transferred separately into WT hosts and infected with LCMV Arm (Fig. 6e). Since TCF-1 is required to generate CD8^+^ memory T cell recall responses^43^, we next investigated the expression of TCF-1 in *Hmgb2*^*−/−*^ P14 memory T cells prior to secondary infection. We found similar TCF-1^+^ frequencies between WT and *Hmgb2*^*−/−*^ P14 T cells both during and after acute Arm infection (Supplementary Fig. 9a). These findings showed that HMGB2 expression was required for the re-expansion of memory CD8^+^ T cells during secondary viral infection.

### *Hmgb2*^*−/−*^ exhausted CD8^+^ T cells are decreased after secondary acute LCMV challenge

We observed significantly decreased frequencies of *Hmgb2*^*−/−*^ Tpex cells during Cl13 infection, and since these cells drive the limited reinvigoration of exhausted T cells after secondary infections^11,12^, we next examined whether exhausted *Hmgb2*^*−/−*^ CD8^+^ T cells could respond to Arm infection. Small numbers (1 × 10^3^ cells/each) of WT and *Hmgb2*^*−/−*^ P14 T cells were adoptively co-transferred at a 1:1 ratio into WT mice and infected with Cl13 (Fig. 6f). At 30dpi, exhausted P14 T cells were sorted from spleens and co-transferred (2 × 10^3^ cells/each) at a 1:1 ratio into new WT mice, which were then infected with LCMV Arm (Fig. 6f). Exhausted WT P14 T cells re-expanded with secondary challenge, while *Hmgb2*^*−/−*^ P14 T cells failed to expand and were at significantly decreased frequencies in the blood (Fig. 6g). Furthermore, we observed significantly decreased frequencies and numbers of *Hmgb2*^*−/−*^ P14 T cells in spleens at 20dpi (Fig. 6h, i). Similar results were seen when exhausted WT and *Hmgb2*^*−/−*^ P14 T cells were transferred separately into WT hosts and re-challenged with LCMV Arm (Fig. 6j). These findings showed HMGB2 expression is essential for the re-expansion of exhausted CD8^+^ T cells after a secondary viral challenge.

### HMGB2 regulates the chromatin accessibility of Tpex and Tex genes during Cl13 infection

**Fig. 7:**
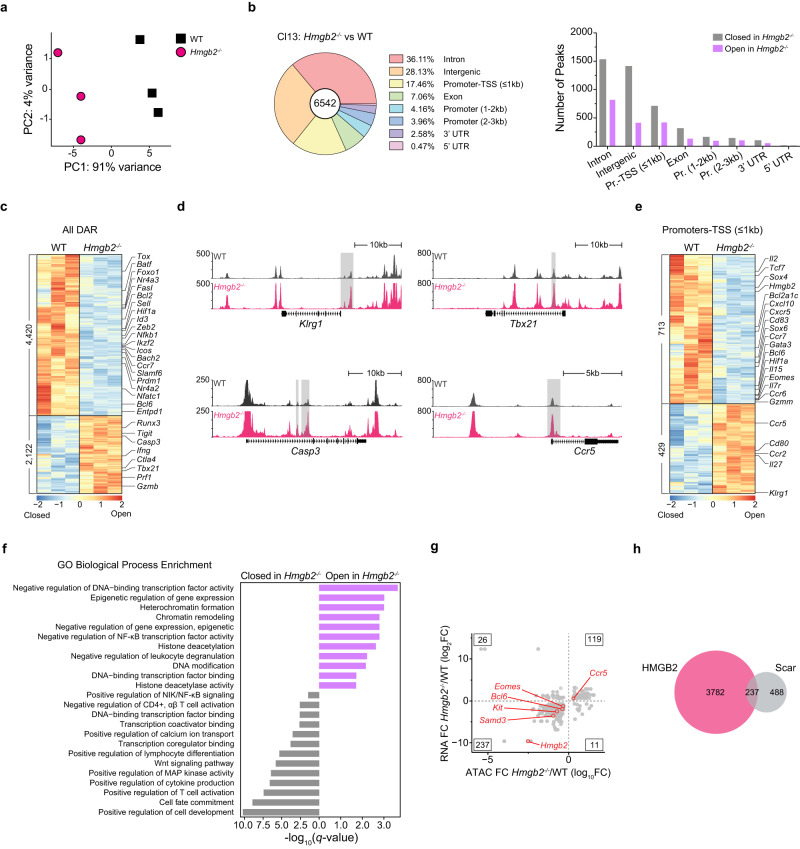
Epigenetic programming of exhausted T cells by HMGB2. ATAC-seq analysis of WT and *Hmgb2*^*−/−*^ P14 T cells at 8dpi LCMV Cl13. **a** Principal component analysis (PCA) of all samples by global chromatin accessibility profile. **b** Location of significantly differentially accessible ATAC-seq peaks (FDR ≤ 0.05, |log_10_FC | ≥ 0.3). **c** Heatmap of all significantly differentially accessible loci (DAR). Numbers on left denote number of DAR. Each column represents a biological replicate of *n* = 10 mice pooled. **d** ATAC-seq tracks of genes associated with effector (Teff) and terminal exhausted (Tex) T cells. DAR are highlighted with gray bars. **e** Heatmap of DAR within promoters-TSS (≤1 kb). Each column represents a biological replicate of *n* = 10 mice pooled. **f** Gene ontology (GO) biological process enrichment from Metascape of DAR within promoters-TSS (≤1 kb) from (**e**). **g** Fold change in ATAC accessibility versus RNA expression. Key genes with DAR in promoters-TSS (≤1 kb) are highlighted in red. Inset, table enumerating number of ATAC peak-gene pairs in each quadrant. **h** Venn diagram of overlap in significant DAR from (**c**) and scarred DAR identified in Abdel-Hakeem et al. ^21^.

Since HMGB2 has a well-characterized role in chromatin remodeling, we next asked whether HMGB2 regulates the epigenetic program of exhausted T cells. We sorted WT and *Hmgb2*^*−/−*^ P14 T cells from mice at 8dpi Cl13 and used ATAC-seq to identify significant changes in chromatin accessibility in the absence of HMGB2. PCA of the ATAC-seq profiles segregated WT and *Hmgb2*^*−/−*^ P14 T cells across PC1 (91% variance), indicating that *Hmgb2* has a significant effect on chromatin accessibility (Fig. 7a). We found 6,542 differentially accessible regions (DAR), with most having decreased accessibility in *Hmgb2*^*−/−*^ P14 T cells compared with WT (Fig. 7b). Genomic annotation showed about 17.5% of these accessibility changes were at promoters (≤1 kb) or transcription start sites (TSS) (Fig. 7b). The genes in close proximity to loci with reduced accessibility in *Hmgb2*^*−/−*^ P14 T cells were associated with Tpex cells, including *Batf, Foxo1, Id3, Ikzf2, Slamf6, Sell* and *Bach2* (Fig. 7c)^39^. Notably, we also observed decreased accessibility near the pro-survival gene *Bcl2*, which is required for Tpex cell survival (Fig. 7c)^44^. This is consistent with the decreased numbers and increased cell death of *Hmgb2*^*−/−*^ Tpex cells observed during Cl13 infection (Fig. 5f, Supplementary Figs. 7a, 8b). In contrast, loci with increased accessibility in *Hmgb2*^*−/−*^ P14 T cells were near genes associated with apoptosis and terminal Tex cells, including *Tigit, Klrg1, Ccr5, Casp3, Ifng, Ctla4, Tbx21, Prf1, Adam8, Lgals3*, and *Gzmb* (Fig. 7c, d)^39^. We observed a similar trend when looking at promoters-TSS (≤1 kb) containing DAR. Promoters of genes associated with Tpex cells had reduced accessibility in *Hmgb2*^*−/−*^ P14 T cells (*Tcf7, Cxcr5, Ccr7, Gata3, Bcl6, Bcl2a1c, Eomes, Il7r*), while promoters of terminal Tex genes were more accessible (*Klrg1*, *Ccr5)* (Fig. 7e)^10,45–47^.

To further characterize genes associated with DAR at promoters-TSS (≤ 1 kb), we performed pathway enrichment. Genes with increased accessibility at promoters in *Hmgb2*^*−/−*^ P14 T cells showed significant enrichment for GO terms associated with (i) negative regulation of DNA-binding transcription factor activity; (ii) heterochromatin formation; and (iii) negative regulation of gene expression (Fig. 7f). Conversely, genes with less accessible promoter regions in *Hmgb2*^*−/−*^ P14 T cells had significant enrichment of GO terms associated with (i) positive regulation of cell development; (ii) positive regulation of T cell activation; and (iii) transcription coregulator/coactivator binding (Fig. 7f). To assess the correlation between chromatin accessibility and gene transcription, we compared these DAR with our 8dpi Cl13 DEG (Fig. 4a, b). Overall, the epigenetic changes induced by HMGB2 corresponded to functionally relevant events, with the majority of DEG having accompanying changes in chromatin accessibility (119 upregulated DEG with increased accessibility, 237 downregulated DEG with decreased accessibility) (Fig. 7g). For instance, the promoters-TSS (≤1 kb) for *Eomes, Bcl6 and Samd3* were less accessible with lower RNA expression in *Hmgb2*^*−/−*^ P14 T cells, while the *Ccr5* promoter was more accessible with increased RNA expression in *Hmgb2*^*−/−*^ P14 T cells (Fig. 7g). We next compared our DARs from *Hmgb2*^*−/−*^ P14 T cells with previously reported epigenetically “scarred” DARs of exhausted CD8^+^ T cells^21^ and found that HMGB2 regulated ~33% of these “scarred’ signatures as early as 8dpi (Fig. 7h). Our data suggests a significant role for HMGB2 in CD8^+^ T cell chromatin accessibility, and more specifically, the opening of genomic regions associated with progenitor cells and the closing of genomic regions associated with terminal cell differentiation. Together, these data contribute to our understanding of the mechanisms by which HMGB2 regulates the development of exhausted CD8^+^ T cells.

### Cell-intrinsic HMGB2 expression in CD8^+^ T cells is required for anti-tumor responses

**Fig. 8:**
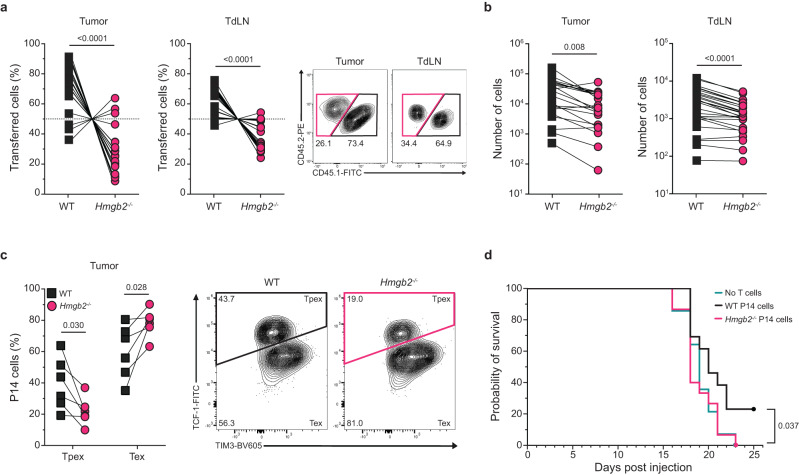
HMGB2 regulation of anti-tumor CD8^+^ T cells. WT and *Hmgb2*^*−/−*^ P14 T cells were co-transferred into WT mice at 1:1 and given B16-GP_33-41_ melanoma cells s.c. Tumors and tumor draining lymph nodes (TdLN) isolated at 18dpi. Frequencies (**a**) and numbers (**b**) of WT and *Hmgb2*^*−/−*^ P14 T cells within the tumor and TdLN at 18dpi; *n* = 21 (tumor), *n* = 27 (TdLN). **c** Frequencies of progenitor exhausted (Tpex) and terminal exhausted (Tex) T cells at 18 dpi in the tumor; *n* = 6. **d** Survival of mice with B16-GP_33-41_ melanoma and adoptive transfer of either WT, *Hmgb2*^*−/−*^, or no P14 T cells; *n* = 14. Data is mean ± s.e.m. Data are representative of three independent experiments except (**a**, **b**, **d**) which are cumulative data from two or more independent experiments. Statistical significance was calculated using a paired two-tailed Student’s *t* test (**a**–**c**) or log-rank (Mantel–Cox) test (**d**). Source data are provided as a Source Data file. (**a**) and (**f**) created with biorender.com.

Since persistent antigen presentation in tumors also drives differentiation of exhausted CD8^+^ T cells, we next asked whether HMGB2 regulated tumor-specific CD8^+^ T cells. We co-transferred congenically marked (1 × 10^6^ cells/each) WT and *Hmgb2*^*−/−*^ P14 T cells at a 1:1 ratio into WT mice and subcutaneously injected B16-GP_33-41_ melanoma cells (1 × 10^6^ cells) a day later. B16-GP_33-41_ melanoma cells are highly aggressive and express the LCMV GP_33-41_ epitope, which is recognized by P14 T cells. We observed significantly decreased frequencies of *Hmgb2*^*−/−*^ P14 T cells compared to WT in the tumor and tumor-draining lymph nodes (TdLNs) at day 18 post melanoma cell injection (Fig. 8a). The numbers of *Hmgb2*^*−/−*^ P14 T cells were also significantly decreased in the tumor and TdLNs (Fig. 8b). We next evaluated the frequencies of Tpex and Tex cells by measuring TCF-1 and TIM-3 expression of tumoral WT and *Hmgb2*^*−/−*^ P14 T cells and found significantly less *Hmgb2*^*−/−*^ Tpex cells compared to WT, similar to our findings during Cl13 infection (Fig. 8c, Fig. 5f, Supplementary Fig. 7a). These data indicate HMGB2 expression is also required for the maintenance and differentiation of Tpex and exhausted CD8^+^ T cells in melanoma tumors.

To assess the role of HMGB2 expression in CD8^+^ T cells on tumor control, we adoptively transferred 1 × 10^6^ congenically marked WT or *Hmgb2*^*−/−*^ P14 T cells into separate, congenically mismatched WT mice. The next day, mice were given 1 × 10^6^ B16-GP_33-41_ melanoma cells s.c. We observed the highest median survival in mice given WT P14 T cells, with mice receiving *Hmgb2*^*−/−*^ P14 T cells or no T cells having similar survival (Fig. 8d). These findings suggest HMGB2 expression in exhausted CD8^+^ T cells is critical for anti-tumor immunity in melanoma tumors.

## Discussion

Our findings showed a cell-intrinsic role for HMGB2 in the differentiation and stemness of memory and exhausted CD8^+^ T cells in viral and tumor models. We found HMGB2 expression and upregulation in effector, memory and exhausted CD8^+^ T cells. During acute viral infection, we found an important role for HMGB2 in memory T cell differentiation of MPEC and Tcm phenotypes, and memory recall responses. In response to chronic viral infection, exhausted *Hmgb2*^−/−^ CD8^+^ T cells showed decreased progenitor exhausted T cell (Tpex) differentiation and survival, with these cells unable to persist during prolonged infection. Despite *Hmgb2*^*−/−*^ CD8^+^ T cells expressing TCF-1 and TOX master regulators, these transcription factors failed to induce the differentiation of Tpex and Tex cells. Transcriptomic and chromatin accessibility analyses revealed that HMGB2 in exhausted CD8^+^ T cells functioned to increase expression and accessibility of Tpex-specific gene signatures, while decreasing expression and chromatin accessibility of Tex gene signatures.

After acute viral infections, effector CD8^+^ T cells differentiate into memory T cells (Tmem), which self-renew, persist long term, and provide protection upon secondary infection with the same pathogen^48–51^. We found that HMGB2 regulated memory CD8^+^ T cell differentiation, as shown by the increased MPEC phenotype in *Hmgb2*^*−/−*^ CD8^+^ T cells. Interestingly, however, we observed increased apoptosis in *Hmgb2*^*−/−*^ MPECs, indicating that HMGB2 promoted the survival of this population. We also found decreased *Hmgb2*^*−/−*^ Tcm cells. Tcm cells are important for Tmem responses as they contribute to their proliferation, longevity, multipotency and recall potential^52,53^. We correspondingly found diminished maintenance and recall capacity of *Hmgb2*^*−/−*^ Tmem cells, which could be attributed to this decrease in Tcm phenotype. Additionally, although *Hmgb2*^*−/−*^ Tmem cells had high expression of the transcription factor TCF-1, a critical regulator for memory T cell transcriptional programs and Tcm-mediated recall responses^43,54,55^, they were still unable to persist and respond to secondary infection. Our findings highlight an important function of HMGB2 in the differentiation, survival, and recall function of memory CD8^+^ T cells.

Compared to effector and memory T cells, the regulation of exhausted CD8^+^ T cells remains poorly understood. Persistent Cl13 infection induces NFAT and calcineurin signaling, which induces TOX expression in CD8^+^ T cells^36,38,56^. We observed similar levels of TOX expressed in WT and *Hmgb2*^*−/−*^ CD8^+^ T cells during Cl13 infection, suggesting effective TCR, NFAT, and calcineurin signaling in *Hmgb2*^*−/−*^ CD8^+^ T cells. Accordingly, both WT and *Hmgb2*^*−/−*^ P14 T cells expanded to similar numbers by 8dpi Cl13 infection, indicating that *Hmgb2*^*−/−*^ P14 T cells were effectively primed and activated during early stages of chronic viral infection. However, despite similar phenotypes and initial responses of exhausted WT and *Hmgb2*^*−/−*^ P14 T cells, *Hmgb2*^*−/−*^ P14 T cells drastically declined after 8dpi Cl13 infection and did not persist. During Cl13 infection, it has been shown that the transcription factors TOX and TCF-1 are critical to form exhausted T cells^2,37^. However, the loss of exhausted *Hmgb2*^*−/−*^ P14 T cells was not due to diminished TOX or TCF-1 expression, as both transcriptional regulators were expressed at similar levels to WT. Importantly, TOX and TCF-1 were insufficient to establish and sustain *Hmgb2*^*−/−*^ Tex cells throughout Cl13 infection. This links HMGB2 proteins with TOX and TCF-1 regulation of exhausted T cell differentiation.

HMGB2 proteins regulate cellular stemness, as shown by defects in various differentiation programs in *Hmgb2*^*−/−*^ mice^57–61^. During chronic infection, stem-like Tpex cells arise, which can self-renew and seed the Tex pool. Using transcriptomics, we found HMGB2 positively regulated the expression of Tpex cell-associated genes and correspondingly, we found HMGB2 positively regulated Tpex cell frequencies, numbers, and survival. HMGB2’s regulation of Tpex cell differentiation and long-term maintenance is similar to that of TCF-1, a key transcriptional regulator of Tpex-specific programming; *Tcf7*^*−/−*^ CD8^+^ T cells fail to develop into Tpex cells and decline throughout Cl13 infection^2,62^. However, despite the loss of *Hmgb2*^*−/−*^ Tpex cells, *Hmgb2*^*−/−*^ T cells had similar frequencies of TCF-1^+^ cells compared to WT. Furthermore, exhausted *Tcf7*^*−/−*^ CD8^+^ T cells have increased expression of *Hmgb2* compared to TCF-1^+^ CD8^+^ T cells^2^. Together, these data suggest HMGB2 and TCF-1 co-regulate Tpex cell differentiation. There is significant clinical interest in understanding the differentiation of Tpex cells for immunotherapy use in cancer and chronically infected patients; Tpex cells provide the effector T cell proliferative burst after immune checkpoint blockade (ICB) and may have therapeutic predictive value in patients^10,15,16,63,64^. Although we did not evaluate the response of *Hmgb2*^*−/−*^ Tpex cells to anti-PD-1/anti-PD-L1 blockade, we observed a defect in their reinvigoration with secondary Arm infection. Therefore, combining HMGB2 modulation with anti-PD-1/anti-PD-L1 therapy may enhance and preserve Tpex differentiation and increase clinical efficacy in the settings of chronic infections and cancer. Together, our findings showed HMGB2 regulates the differentiation, survival, and reinvigoration of Tpex cells, and may help predict ICB efficacy.

Exhausted T cells develop permanent epigenetic marks early in their differentiation, with additional epigenetic changes acquired at later stages of exhaustion^4,5,8^. Since the epigenetic program of exhausted T cells is relatively stable, ICBs can only transiently reinvigorate exhausted T cells, as they reacquire their exhausted epigenetic program over time^11,18,21–23^. Combining chromatin remodeling with ICBs may represent a new clinical approach to increase the reinvigoration potential of exhausted T cells. Therefore, identifying exhaustion-specific epigenetic regulators is a pressing clinical need for patients with chronic diseases. HMGB2 is a known chromatin modifier, but its role in the chromatin remodeling and epigenetic programming of exhausted T cells is not known. Here, we found that HMGB2 regulated the accessibility of genomic regions in exhausted T cells, with most of these changes corresponding to functionally relevant events in gene transcription. HMGB2 directly supported the accessibility of Tpex associated genes, many of which are regulated by the transcription factor TCF-1, while decreasing the accessibility of genes associated with Tex cells and apoptosis. Therefore, we hypothesize that HMGB2 supports Tpex cell differentiation by modulating the accessibility and expression of genes regulated by TCF-1 and possibly other critical transcriptional regulators. Notably, Ingenuity Pathway Analysis (IPA) identified the TCF-1 master regulatory pathway as being one of many significantly inhibited in *Hmgb2*^*−/−*^ P14 T cells, suggesting HMGB2 is required for TCF-1 mediated transcriptional programs in exhausted T cells. HMGB2 may enhance TCF-1 binding to its targets, as it does with LEF-1, a transcription factor functionally similar to TCF-1^60,65^. Furthermore, TCF-1 and LEF1 have been shown to regulate CD8^+^ T cell identity and function, with ablation of these transcription factors resulting in expression of non-T cell lineage genes^66^. Although exhausted *Hmgb2*^*−/−*^ P14 T cells had high TCF-1 expression, we found similar aberrant T cell gene expression at 20dpi Cl13 (*Cd19, Cd4*, and immunoglobulins). We propose that HMGB2 and TCF-1 co-regulate exhaustion-specific transcriptional and epigenetic programs in CD8^+^ T cells through chromatin remodeling and facilitating transcription factor binding.

In summary, we show that *Hmgb2* expression is required for the differentiation and survival of memory and Tpex cells during acute and chronic viral infection, respectively. We detected HMGB2 expression in naïve, effector, memory, and exhausted T cells after LCMV infection in mice, with higher levels during persistent infection. In the setting of acute viral infection, *Hmgb2*^*−/−*^ memory CD8^+^ T cells developed, but they were defective in their differentiation and recall capacity. During chronic Cl13 infection, *Hmgb2*^*−/−*^ CD8^+^ T cells initially proliferated and expanded to similar levels as WT but were severely hindered in their formation of Tpex cells, thus preventing long-term exhausted T cell responses. We found HMGB2 increased the accessibility of signature genes which promoted the transcriptional programming, differentiation, and maintenance of Tpex cells. We also observed decreased *Hmgb2*^*−/−*^ Tpex cells in melanoma tumors and tumor-draining lymph nodes, indicating HMGB2 sustains exhausted T cells in multiple models of persistent antigen. We found a previously unidentified role for HMGB2 in the differentiation and survival of functional memory and exhausted T cells, with vast implications for secondary reinfections and immunotherapies to cancer and chronic viruses. This understanding of HMGB2’s role in exhausted T cell stemness and its contribution to TCF-1 and TOX mediated regulation of exhaustion shows that HMGB2 is an indispensable partner of TCF-1 and TOX in the formation and maintenance of exhausted T cells.

## Methods

### Reagent

For a complete list of reagents, see Supplementary Table 1

### Mice

All experimental animal procedures were approved by the Institutional Animal Care and Use Committee of University of California, Irvine (AUP-21-124) and complied with all relevant ethical regulations for animal testing and research. Mice were housed in an animal facility at UCI on a 12-hour light/12-hour dark cycle at ~72°F and ~55% humidity. C57BL/6J and B6.SJL-*Ptpr*^*a*^ *Pepc*^*b*^/BoyJ mice were purchased from the Jackson Laboratory, then bred in SPF facilities. P14 mice were obtained from The Scripps Research Institute (originally from Dr. Charles D. Surh). These mice were bred to Ly5.1 (B6.SJL-*Ptprc*^*a*^ *Pepc*^*b*^/BoyJ) mice and to *Hmgb2*^*−/−*^ mice, which were generously provided by Dr. Marco Bianchi (San Raffaele Scientific Institute, Milan, Italy). Male and female mice ≥ 6 weeks of age were used in experiments. Mouse selection for experiments was not formally randomized or blinded.

### Virus infection and titers

LCMV Armstrong (Arm) and Clone 13 (Cl13) strains were propagated in baby-hamster kidney cells and titrated on Vero African-green-monkey kidney cells. Frozen stocks were diluted in Vero cell media and 2 × 10^5^ plaque-forming units (PFUs) of LCMV Arm were injected intraperitoneally (i.p.) and 2 × 10^6^ PFUs of LCMV Cl13 were injected intravenously (i.v.). Virus titers were determined from serial dilutions of either sera or tissues taken from mice using a plaque assay.

### T cell adoptive transfer

Bulk CD8^+^ T cells were enriched from spleens and lymph nodes (LNs) of WT (*Hmgb2*^+/+^) or *Hmgb2*^−/−^ P14 transgenic mice by column-free magnetic negative selection. Single-cell suspensions from pooled spleen and LNs were incubated with biotinylated antibodies against CD4 (GK1.5), B220 (RA3-6B2), CD19 (6D5), CD24 (M1/69), CD11b (M1/70), and CD11c (N418). Non-CD8^+^ cells were removed by mixing labeled cell suspension with Streptavidin RapidSpheres (Stemcell technologies) at room temperature (RT) for 5 min, followed by two-5 min incubations in an EasyEights EasySep Magnet (Stemcell technologies). The unbound CD8^+^ T cells were washed in sterile PBS (1x) with FBS (2%), and purity was determined on a flow cytometer. For single-transfer studies, WT and *Hmgb2*^−/−^ P14 T cells were transferred into separate new WT hosts of the opposite congenic marker (1 × 10^3^ i.v. for virus studies, 1 × 10^6^ for tumor survival studies). For co-transfer studies, WT and *Hmgb2*^−/−^ P14 T cells were mixed at a 1:1 ratio (1 × 10^3^ i.v. per cell-type for virus studies, 1 × 10^6^ i.v. per cell type for tumor studies) and injected into new WT recipient mice i.v. Within 18–24 h post-transfer, recipient mice were inoculated with LCMV Arm (2 × 10^5^, i.p.), LCMV Cl13 (2 × 10^6^ PFU, i.v.), or B16-GP_33-41_ tumor cells (1 × 10^6^, s.c.). For re-challenge experiments, live (PI^−^) WT and *Hmgb2*^−/−^ P14 T cells were sorted at >95% purity from spleens and LNs at 30dpi or 68dpi using a BD FACSAria sorter. Cell numbers were normalized and transferred into new hosts (2 × 10^3^ i.v. per cell-type) that were subsequently infected with LCMV Arm (2 × 10^5^ PFU, i.p.).

### B16-GP_33-41_ tumor model and digestion

B16-GP_33-41_ melanoma cells were kindly provided by Dr. Ananda Goldrath. All cell lines maintained in Iscove’s Modified Dulbecco’s medium supplemented with 10% fetal bovine serum (FBS) and antibiotics. All cell lines were free of mycoplasma. For co-transfer and survival tumor experiments, mice were injected subcutaneously (s.c.) with 1 × 10^6^ tumor cells. Tumor size was measured by caliper daily for calculation of B16-GP_33-41_ tumor volume and tumors of <1800mm^3^ were designated as surviving per IACUC protocol. Tumors were weighed at time of excision before being minced and digested in gentleMACS C Tubes for 40 min at 37 °C using the gentleMACS Dissociator (Miltenyi). Digests were then passed through a 70-μm cell strainer to generate a single-cell suspension. The cells were then stained for flow cytometry.

### Antibodies

The following fluorochrome-conjugated antibodies were used (clone mentioned in parentheses). From Abcam: anti-HMGB2 unconjugated (ERP6302), dilution 1:100. From BD: anti-Ly108-BV421 (13G3), 1:200; anti-Ly108-PE (13G3), 1:200; anti-TCR Vß8.1.2-FITC (MR5-2), 1:200. From BioLegend: anti-Bcl6-APC (7D1), dilution 1:200; anti-CCR7-BV605 (4B12), 1:200; anti-CCR7-PE (4B12), 1:200; anti-CD127-BV605 (A7R34), 1:200; anti-CD223-PerCP5.5 (C9B7W), 1:200; anti-CD279-BV510 (29F.1A12), 1:200; anti-CD279-PE-Cy7 (29F.1A12), 1:200; anti-CD44-APC-Cy7 (IM7), 1:200; anti-CD44-PE (IM7), 1:200; anti-CD45.1-APC (A20), 1:200; anti-CD45.1-FITC (A20), 1:200; anti-CD45.1-Pacific Blue (A20), 1:200; anti-CD45.1-PE-Cy7 (A20), 1:200; anti-CD45.2-APC (104), 1:200; anti-CD45.2-APC-Cy7 (104), 1:200; anti-CD45.2-BV605 (104), 1:200; anti-CD45.2-FITC (104), 1:200; anti-CD45.2-Pacific Blue (104), 1:200; anti-CD45.2-PE (104), 1:200; anti-CD45.2-PE-Cy7 (104), 1:200; anti-CD62L-PE (MEL-14), 1:200; anti-CD62L-PerCP (MEL-14), 1:200; anti-CD69-PE-Cy7 (H1.2F3), 1:200; anti-CD8α-APC (53-6.7), 1:200; anti-CD8α-BV510 (53-6.7), 1:200; anti-CD8α-BV605 (53-6.7), 1:200; anti-CD8α-BV785 (53-6.7), 1:200; anti-CD8α-Pacific Blue (53-6.7), 1:200; anti-CD8α-PE (53-6.7), 1:200; anti-CD8α-PE-Cy7 (53-6.7), 1:200; anti-CXCR5-APC-Cy7 (L138D7), 1:200; anti-IFN-γ-APC (XMG1.2), 1:100; anti-IFN-γ-FITC (XMG1.2), 1:100; anti-IL-2-PE (JES6-5H4), 1:100; anti-KLRG1-APC (2F1/KLRG1), 1:200; anti-KLRG1-FITC (2F1/KLRG1), 1:200; anti-TCR Vα2-PE (B20.1), 1:200; anti-TNF-PE-Cy7 (MP6-XT22), 1:100; Biotin anti-CD11b (M1/70), 1:30; Biotin anti-CD11c (N418), 1:30; Biotin anti-CD16/32 (93), 1:30; Biotin anti-CD19 (6D5), 1:30; Biotin anti-CD24 (M1/69), 1:30; Biotin anti-CD4 (GK1.5), 1:30; Biotin anti-CD45R/B220 (RA3-6B2), 1:30; Donkey anti-rabbit IgG-AF488 (Poly4064), 1:200; Donkey anti-rabbit IgG-AF647 (poly4064), 1:200. From Cell Signaling: anti-TCF1/TCF7-AF488 (C63D9), 1:100; anti-TCF1/TCF7-Pacific Blue (C63D9), 1:100. From Fisher: Granzyme B (GB12), 1uL/well; Ki-67 FITC (B56), 1:20; 7-AAD, 1:20. From Miltenyi: anti-TOX-PE (REA473), 1:100. From National Institute of Health (NIH) tetramer core: H-2Db-GP33-41 tetramer, 1:200; H-2Db-GP276-286 tetramer, 1:200; H-2Db -NP396-404 tetramer, 1:200.

### Flow cytometry

For cell surface staining, 2 × 10^6^ cells were incubated with antibodies in staining buffer (PBS, 2% FBS and 0.01% NaN_3_) at 4 °C. For tetramer surface staining, 2 × 10^6^ cells were stained with conjugated H-2D^b^-GP_33–41_, H-2D^b^-GP_276-286_, or H-2D^b^-NP_396–404_ tetramers (NIH core facility) for 1 h and 15 min at RT in staining buffer. For intracellular cytokine staining, cells were resuspended in complete RPMI-1640 (containing 10 mM HEPES, 1% nonessential amino acids and L-glutamine, 1 mM sodium pyruvate, 10% heat inactivated FBS and antibiotics) supplemented with 50 U/mL IL-2 (NCI) and 1 mg/mL brefeldin A (BFA, Sigma), and then incubated with 2 mg/mL LCMV GP_33-41_ peptide (AnaSpec) at 37 °C for 4 h. Cells were then fixed and permeabilized using a Cytofix/Cytoperm Kit (BD Biosciences) before staining. For intranuclear transcription factor staining, cells were fixed and permeabilized using a Foxp3/transcription factor fixation/permeabilization kit (Fisher). Surface stains were performed at a 1:200 dilution, while intracellular and intranuclear stains performed at a 1:100 dilution. Caspase3 staining was done using CaspGLOW Fluorescein Active Caspase-3 staining kit (ThermoFisher) following manufacturer’s instructions. All data were collected on a Novocyte3000 (Agilent) and analyzed using FlowJo v10.9.1 (Tree Star).

### Imaging flow cytometry

For imaging flow cytometry, negative selection was performed (above) to isolate CD8^+^ T cells and cells were stained as described previously. Zombie staining was done using Zombie Aqua Fixable Viability Kit (BioLegend) as outlined by manufacturer’s instructions. 7-ADD (Fisher) was used to stain nuclei per manufacturer’s instructions. Cells were resuspended at 2 × 10^7^ cells/mL and run on an Amnis ImageStream X Mark II imaging flow cytometer (EMD Millipore) and analyzed using IDEAS software (EMD Millipore).

### In vivo proliferation

Mice were injected i.p. with 2 mg BrdU (Sigma-Aldrich) 16 h before removing spleens at 8dpi to measure proliferation. Cells were stained intracellularly using FITC BrdU Flow kit (BD Biosciences) following the manufacturer’s instructions. Cells were acquired with a Novocyte3000 flow cytometer.

### Comet assay

Co-transferred live (PI^-^) WT and *Hmgb2*^−/−^ P14 T cells were sorted at >95% purity from spleens and LNs of LCMV Arm or Cl13 infected mice at 8dpi using a BD FACSAria sorter. U2OS cells were cultured in DMEM supplemented with 10% fetal bovine serum, 1% l-Glutamine, and 1% penicillin/streptomycin. Single‐cell alkaline gel electrophoresis was performed with comet assay Kit (Abcam) following the manufacturer’s instructions. Images were captured using a Leica DMi8 THUNDER microscope. Comet olive tail moments of 100 cells were analyzed using CometScore software version 2.0.0.38.

### Western Blot

Co-transferred live (PI^-^) WT and *Hmgb2*^−/−^ P14 T cells were sorted at >95% purity from spleens and LNs of LCMV Arm or Cl13 infected mice at 8dpi using a BD FACSAria sorter. Cells were lysed and blots were stained for Phospho-H2AX (Ser139) (1:1000) and Histone H3 (1:140000).

### Bulk RNA-Seq RNA isolation and library preparation

For 8dpi studies, WT and *Hmgb2*^−/−^ P14 T cells were transferred into five separate mice each before infection with either LCMV Arm or Cl13. For 20dpi studies, WT and *Hmgb2*^−/−^ P14 T cells were co-transferred at 1:1 into 10 mice before infection with LCMV Cl13. On 8dpi or 20dpi, spleens and LNs were pooled based on infection type and P14 genotype. Live (PI^-^) WT and *Hmgb2*^−/−^ P14 were sorted at >95% purity (8dpi:~1 × 10^6^ per condition; 20dpi: ~300k WT, ~100k *Hmgb2*^−/−^) and resuspended in RLT Buffer and BME before storage at −80 °C. Each experiment was performed three times to represent three biological replicates. Total RNA was monitored for quality control using the Agilent Bioanalyzer Nano RNA chip and Nanodrop absorbance ratios for 260/280 nm and 260/230 nm. Library construction was performed according to the Illumina TruSeq mRNA stranded protocol. The input quantity for total RNA within the recommended range and mRNA was enriched using oligo dT magnetic beads. The enriched mRNA was chemically fragmented. First-strand synthesis used random primers and reverse transcriptase to make cDNA. After second strand synthesis the ds cDNA was cleaned using AMPure XP beads and the cDNA was end repaired and then the 3′ ends were adenylated. Illumina barcoded adapters were ligated on the ends and the adapter ligated fragments were enriched by nine cycles of PCR. The resulting libraries were validated by qPCR and sized by Agilent Bioanalyzer DNA high-sensitivity chip. The concentrations for the libraries were normalized and then multiplexed together. The multiplexed libraries were sequenced using paired-end 100 cycles chemistry on the NovaSeq 6000.

### Bulk RNA-Seq data analysis

Post-processing of the run to generate FASTQ files was performed at the Institute for Genomics and Bioinformatics (UCI IGB). *PcaHubert* was used to identify any outlier samples, which were removed from further analysis^67^. The quality of the sequencing was first assessed using the *fastQC* tool (v0.11.9). Raw reads were then quality trimmed and filtered by a length of 20 bases using *trimmomatic* (v0.39). Trimmed reads were analyzed with the mouse Grcm38 reference genome using pseudo aligner Salmon (v1.2.1) and resulting quantification files were imported using R package *tximport* to get TPM values for all annotated mouse genes. Differential analysis was done using R package *DESeq2* (v1.22.2) with an FDR cut off of 0.05. PCA was done using R packages *DESeq2* and *pheatmap*. For downstream analysis, genes with adjusted *p*-value ≤ 0.1 and |log_2_FC | ≥ 0.5 were included. Gene ontology functional enrichment of gene expression changes in WT and *Hmgb2*^*−/−*^ P14 T cells were performed using Metascape (http://metascape.org).

### ATAC-Seq library preparation

WT and *Hmgb2*^−/−^ P14 T cells were co-transferred at 1:1 into 10 mice before infection with LCMV Cl13. On 8dpi, spleens and LNs were pooled (samples are pooled from 10mice/group). Live (PI^-^) WT and *Hmgb2*^−/−^ P14 were sorted at >95% purity (2 × 10^5^ WT, 2 × 10^5^ *Hmgb2*^−/−^). Each experiment was performed three times to represent three biological replicates. Following the Omni-ATAC protocol, samples were lysed in lysis buffer (10 mM Tris-HCl (pH 7.4), 10 mM NaCl, 3 mM MgCl_2_, 10% Np-40, 10% Tween, and 1% Digitonin) on ice for 3 min^68^. Immediately following lysis, nuclei were spun at 500 g for 10 min at 4 °C to remove supernatant. Nuclei were then incubated with Tn5 transposase for 30 min at 37 °C. Tagmented DNA was purified using AMPure XP beads and PCR was performed to amplify the library under the following conditions: 72 °C for 5 min; 98 °C for 30 s; 5 cycles of 98 °C for 10 s, 63 °C for 30 s, and 72 °C for 1 min; hold at 4 °C. Libraries were then purified with warm AMPure XP beads and quantified on a Bioanalyzer. Libraries were multiplexed and sequenced to a depth of 50million 100 bp paired reads on a NextSeq 500.

### ATAC-Seq data analysis

Paired ended reads from sequencing were QC analyzed with fastqQC (v.11.9) and aligned to mouse mm10 reference genome using bowtie2 (v2.4.1). Mitochondrial reads and reads mapped to dark list (ENCODE Stanford version) were excluded from the downstream analysis. Duplicated reads were removed using Picard tools (v2.27.1). A union peak list was created by merging processed reads from all samples and then calling peaks using MACS2 (v2.7.1) (-q 0.01 --keep-dup all -f BAMPE). The number of reads in each peak were then counted using featureCounts (Rsubread v2.6.4) to create a counts matrix. Normalization of counts matrix was performed using DESeq2 (v1.32.0). Differentially expressed peaks were determined using edgeR (v3.34.1) with an FDR cut-off of 0.05 and a |log_10_FC| cut-off of ≥ 0.3. Peaks were annotated using ChIPSeeker (v1.34.0). Functional enrichment of promoter regions was performed using Metascape (http://metascape.org).

### Quantification and statistical analysis

Flow cytometry data were analyzed with FlowJo v10.9.1 (Tree Star). Bulk RNA-seq and ATAC-seq figures were prepared using RStudio software. Graphs were prepared with Prism 9 (GraphPad Software). Prism was used for statistical analysis to compare outcomes using a two-tailed unpaired Student’s *t* test, Mann–Whitney or two-tailed paired Student’s *t* test where indicated; significance was set to *P* ≤ 0.05. Error bars show SEM.

### Reporting summary

Further information on research design is available in the Nature Portfolio Reporting Summary linked to this article.

### Supplementary information

Supplementary Information

Reporting Summary

### Source data

Source Data

## Data Availability

The authors declare that all supporting data are available within the article, Supplementary Information or from the author upon request. Source data are provided with this paper. RNA-seq and ATAC-seq data have been deposited in the NCBI Gene Expression Omnibus (GEO) database and are accessible through the GEO SuperSeries accession number: GSE237813. Source data are provided with this paper.
