# Supplementary material for: HMGB2 regulates the differentiation and stemness of exhausted CD8+ T cells during chronic viral infection and cancer
Source: Nat Commun. 2023 Sep 13;14:5631. doi: 10.1038/s41467-023-41352-0 (PMC10499904; doi:10.1038/s41467-023-41352-0)
Supplement: Supplementary file 1 — Supplementary Information [file 41467_2023_41352_MOESM1_ESM.pdf]

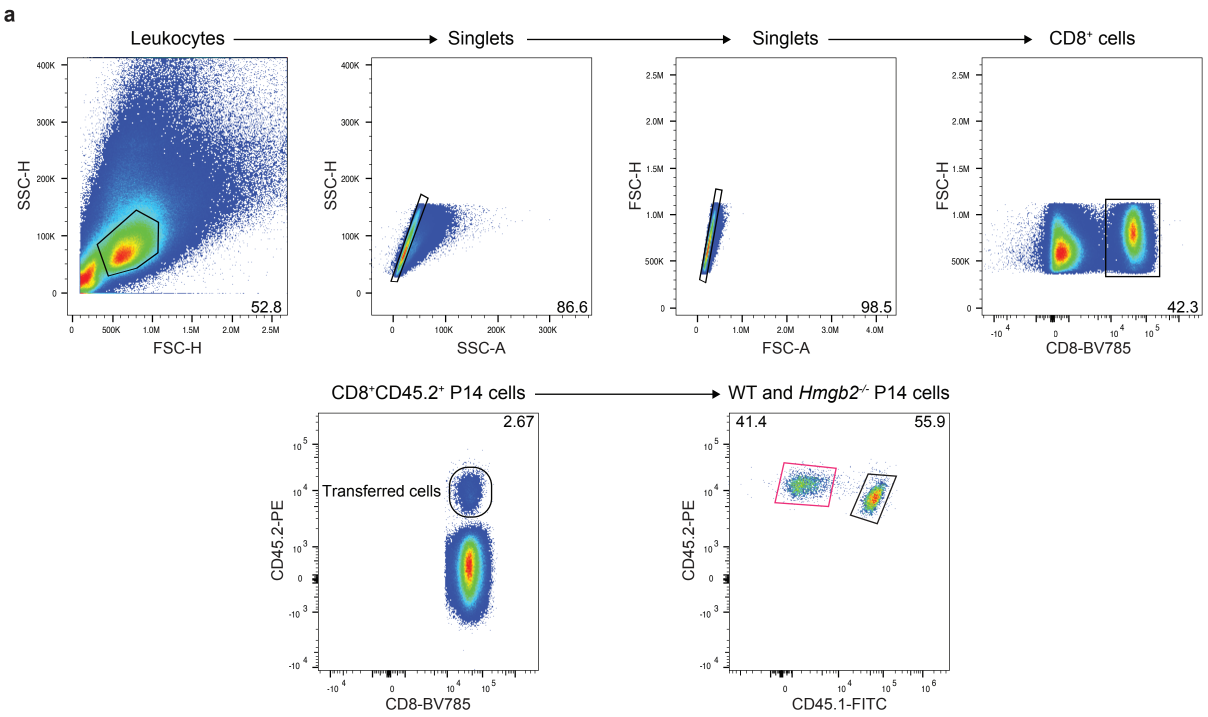

Supplementary Figure 1. Gating strategy

(**a**) Gating strategy used in co-adoptive transfer experiments.

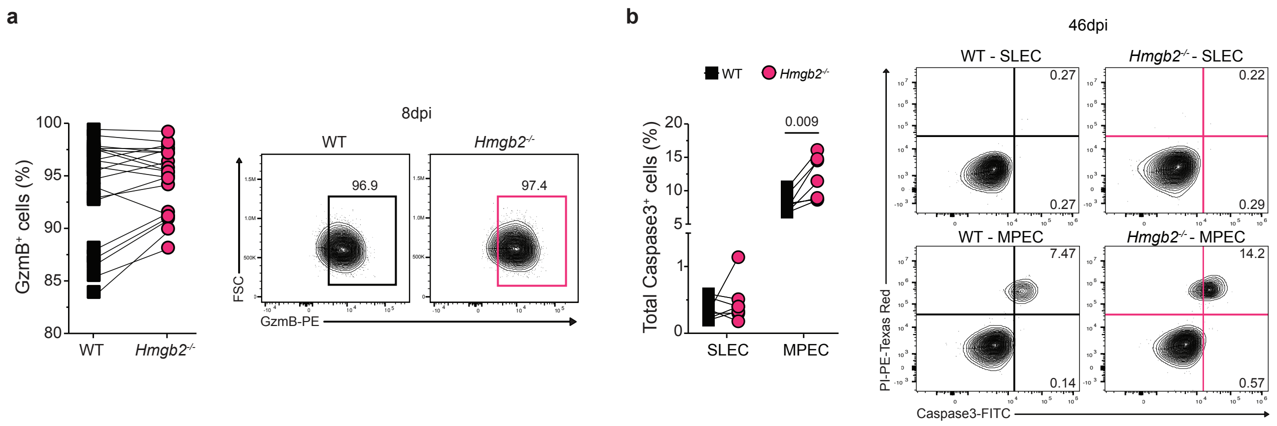

Supplementary Figure 2. Function and differentiation of *Hmgb2*$^{-/-}$ CD8^{+} T cells during acute LCMV infection

(**a**) Frequencies of GranzymeB^{+} WT and *Hmgb2*$^{-/-}$ P14 T cells at 8dpi LCMV Arm in the blood; *n* = 20. (**b**) Frequency of total Caspase3^{+} splenic WT and *Hmgb2*$^{-/-}$ short-lived effector (SLEC) and memory precursor effector (MPEC) P14 T cells at 46dpi Arm; *n* = 7. Data is mean ± s.e.m. Data are representative of three independent experiments except (a) which is cumulative data from three independent experiments. Statistical significance was calculated using a paired two-tailed Student's *t*-test. Source data are provided as a Source Data file.

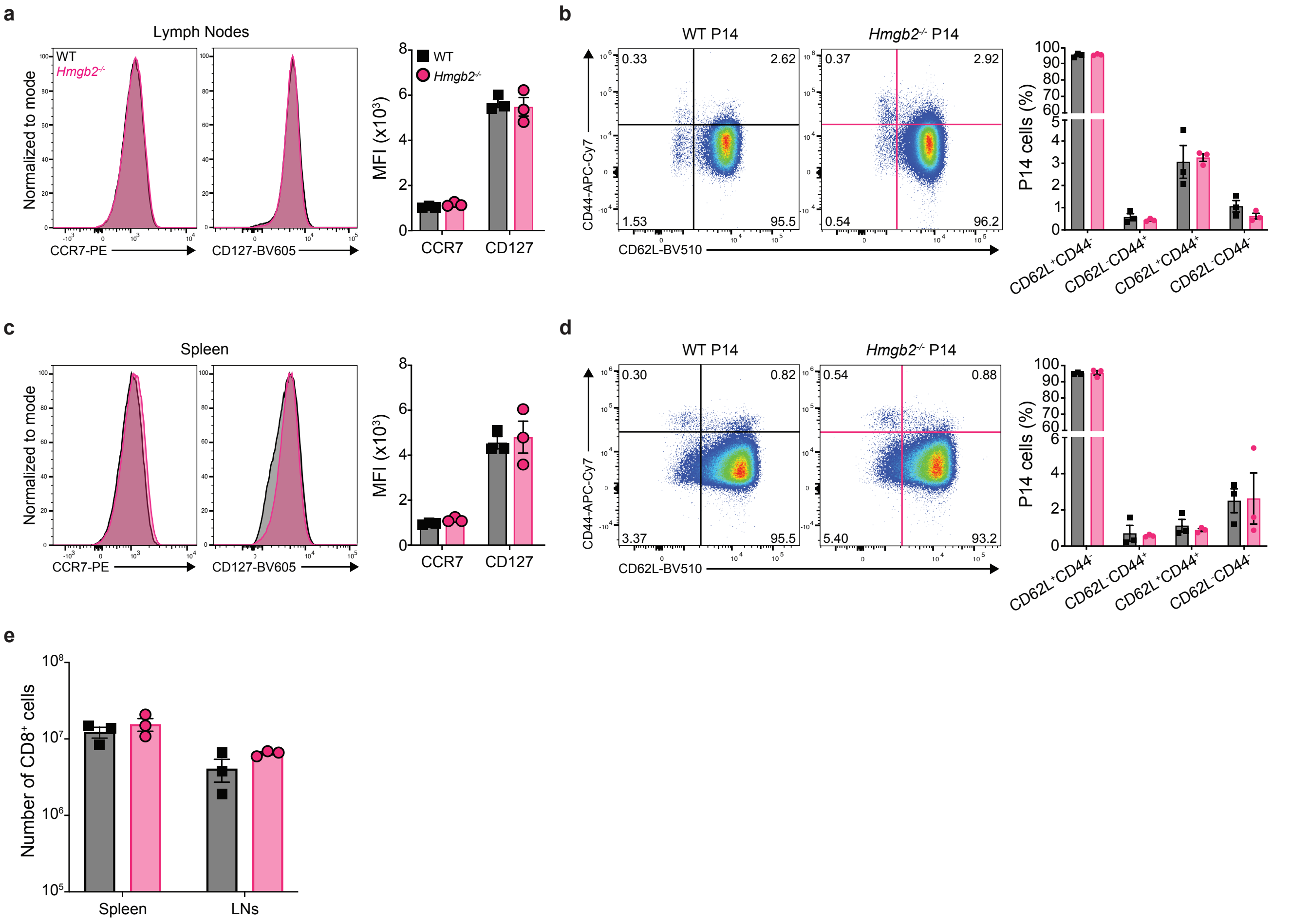

Supplementary Figure 3. HMGB2 expression is dispensable for naïve CD8$^+$ T cell development

CCR7/CD127 (**a**) and CD44/CD62L (**b**) protein expression of naïve WT and *Hmgb2*$^{-/-}$ P14 T cells isolated from lymph nodes of uninfected mice; *n* = 3. CCR7/CD127 (**c**) and CD44/CD62L (**d**) protein expression of naïve WT and *Hmgb2*$^{-/-}$ P14 T cells isolated from spleens of uninfected mice; *n* = 3. (**e**) Number of naïve WT and *Hmgb2*$^{-/-}$ CD8$^+$ T cells; *n* = 3. Data is mean ± s.e.m. Data are representative of three independent experiments. Statistical significance was calculated using an unpaired two-tailed Student's *t*-test followed by Mann-Whitney test. Source data are provided as a Source Data file.

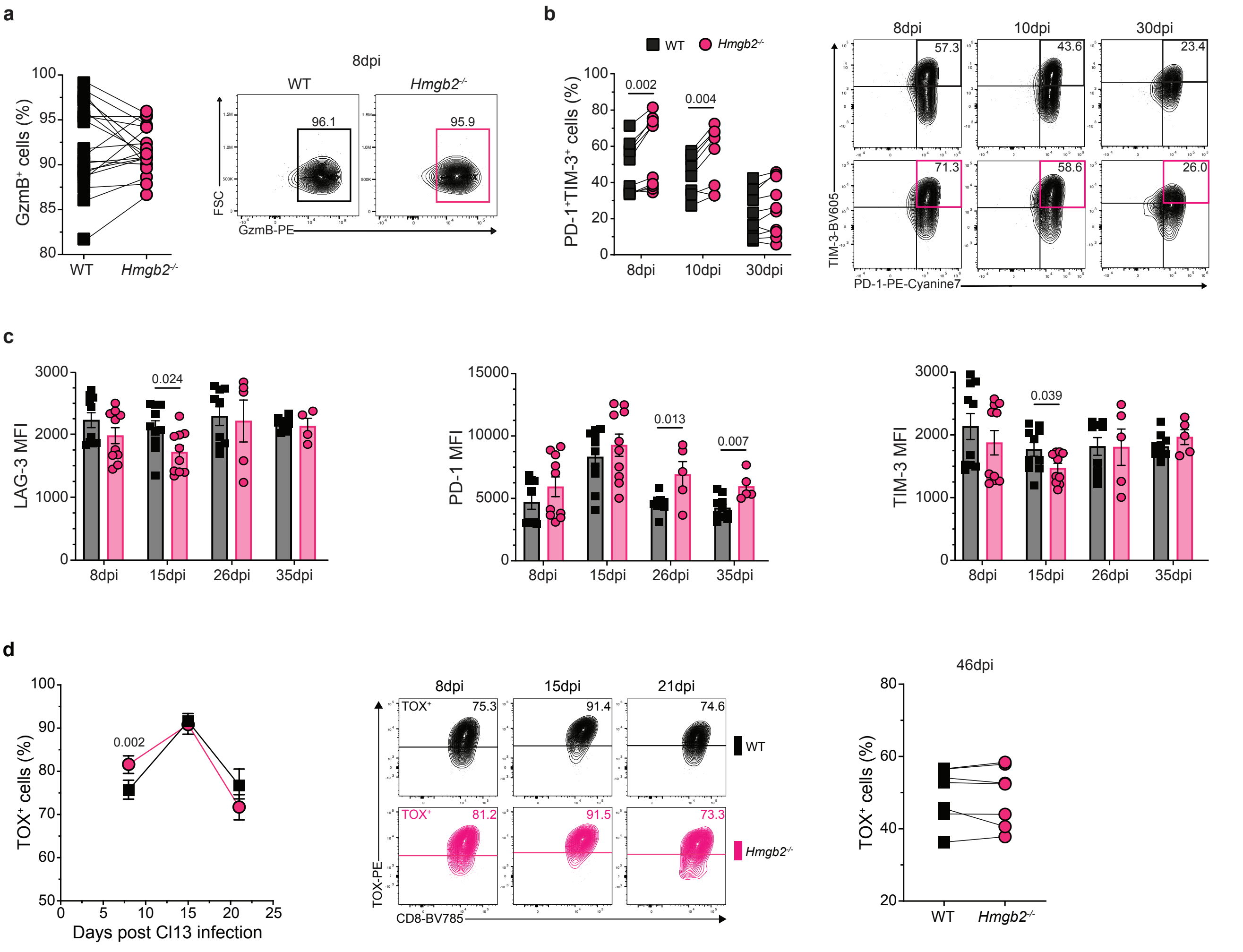

Supplementary Figure 4. Terminal exhaustion of *Hmgb2*$^{-/-}$ CD8$^+$ T cells during chronic LCMV infection

(**a**) Frequencies of GranzymeB$^+$ WT and *Hmgb2*$^{-/-}$ P14 T cells at 8dpi LCMV Cl13 in the blood; *n* = 20. Inhibitory-receptor expression in WT and *Hmgb2*$^{-/-}$ P14 T cells during Cl13 infection in spleens (**b**) and blood (**c**); *n* = 10. (**d**) TOX expression during LCMV Cl13 infection in the blood (8-21dpi) and spleen (46dpi); *n* = 5. Data is mean ± s.e.m. Data are representative of three independent experiments except (a, b, c) which are cumulative data from two or more independent experiments. Statistical significance was calculated using a paired two-tailed Student's *t*-test (**a-b**, **d**) or an unpaired two-tailed Student's *t*-test followed by Mann-Whitney test (**c**). Source data are provided as a Source Data file.

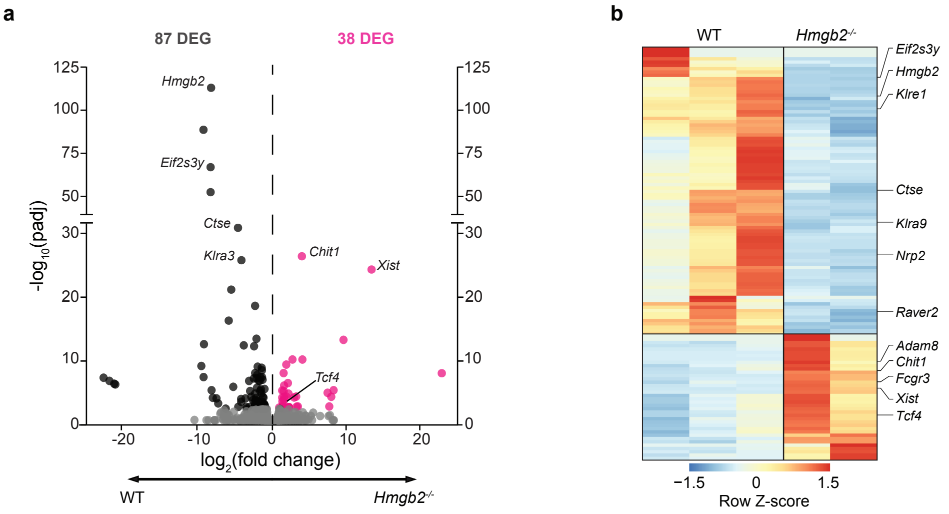

Supplementary Figure 5. Transcriptomics of WT and *Hmgb2*$^{-/-}$ CD8^{+} T cells after acute viral infection

(**a**) Volcano plot highlighting differentially expressed genes (DEG) between WT and *Hmgb2*$^{-/-}$ P14 T cells at 8dpi LCMV Arm infection from Fig. 4a. Significant DEG (padj ≤ 0.1) were determined using *DESeq2* and are colored (pink = upregulated in *Hmgb2*$^{-/-}$ P14 T cells; black = upregulated in WT P14 T cells). (**b**) Heatmap of average normalized expression of significant DEG. Each column represents one independent experiment with *n* = 5 mice pooled.

a

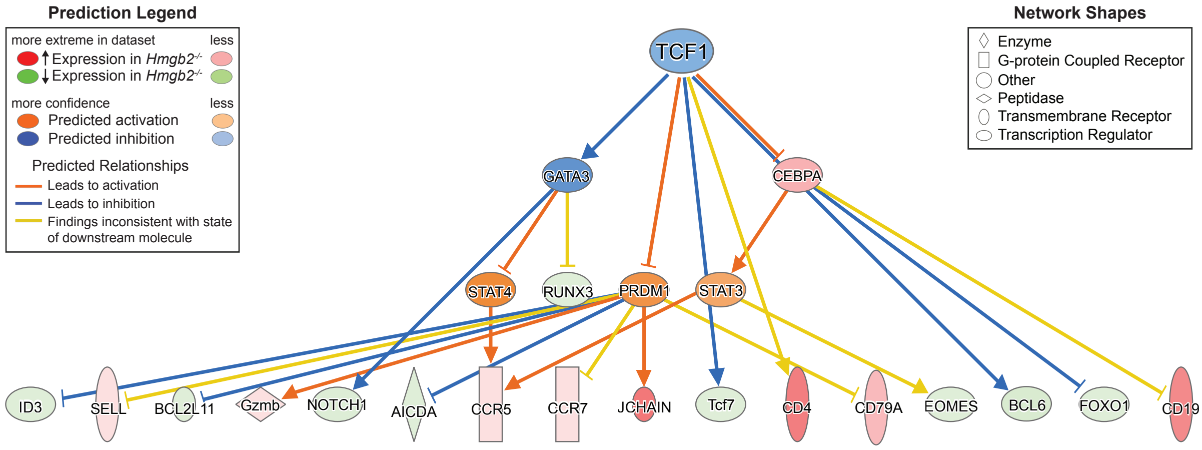

Supplementary Figure 6. HMGB2 regulates TCF-1 transcriptional network in CD8[+] T cells during Cl13 infection

(**a**) TCF-1 causal network identified with Ingenuity Pathway Core Analysis (IPA) (activation z-score = -3.236, network bias-corrected *p*-value = 0.00001). Input was differentially expressed genes (DEG) (padj ≤ 0.1, $|\log_2 FC| \geq 0.5$) between WT and *Hmgb2*$^{-/-}$ P14 T cells at 20dpi Cl13.

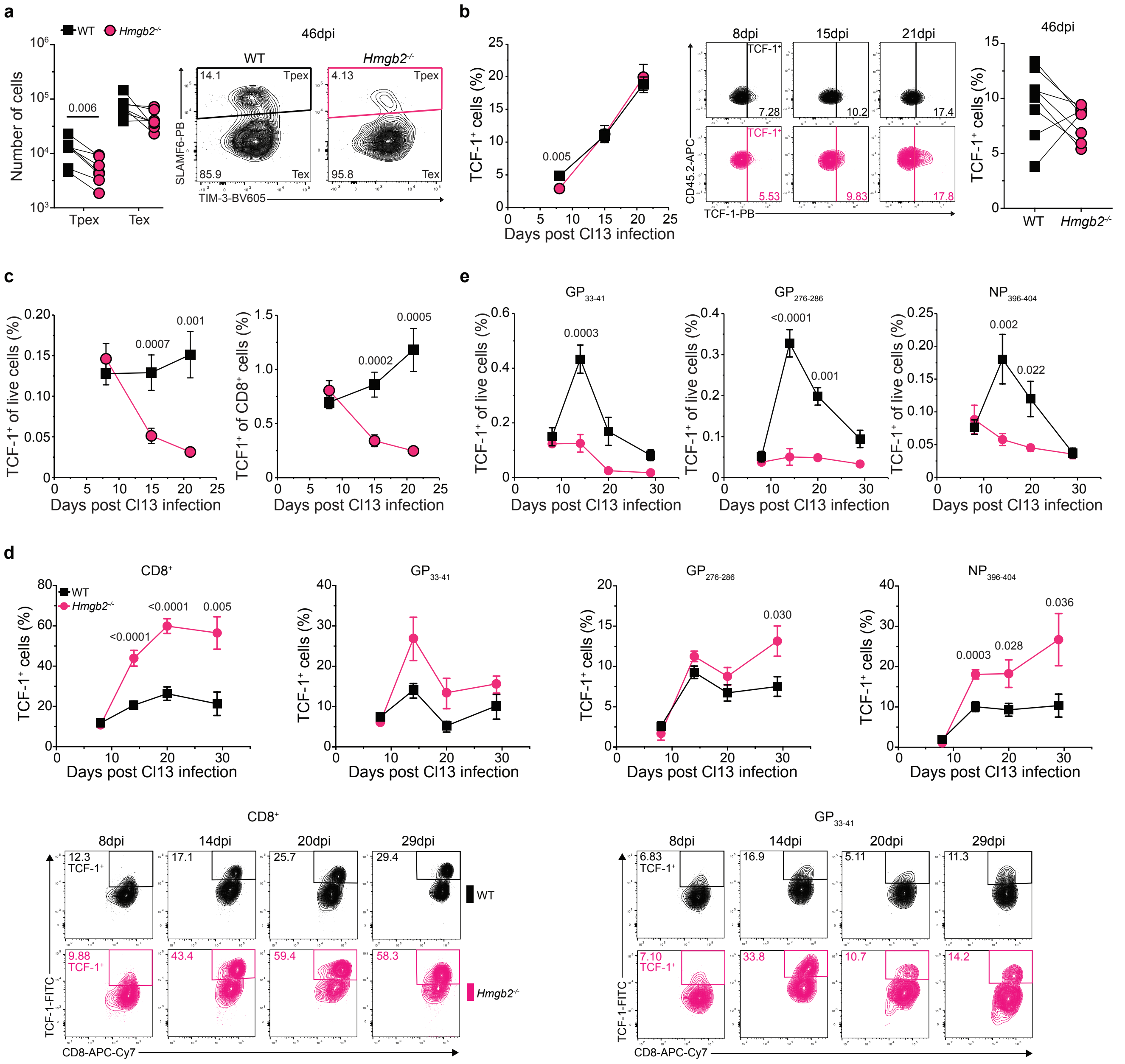

Supplementary Figure 7. TCF-1 expression in exhausted *Hmgb2*$^{-/-}$ CD8$^+$ T cells (**a**) Number of splenic WT and *Hmgb2*$^{-/-}$ P14 progenitor exhausted (Tpex) and terminal exhausted (Tex) T cells at 46dpi Cl13; *n* = 7. (**b**) TCF-1 expression in transferred P14 T cells during Cl13 infection in the blood (8-21dpi) and spleen (46dpi); *n* = 8. (**c**) TCF-1 expression in transferred P14 T cells represented as frequency of live cells (left) and total CD8$^+$ T cells (right) during Cl13 infection in the blood; *n* = 10. (**d**) TCF-1 expression in WT and *Hmgb2*$^{-/-}$ mice during Cl13 infection in the blood; *n* = 9. (**e**) TCF-1 expression in antigen-specific CD8$^+$ T cells in WT and *Hmgb2*$^{-/-}$ mice represented as frequency of live cells during Cl13 infection in the blood; *n* = 9. Data is mean ± s.e.m. Data are representative of two or more independent experiments except (d, e) which are cumulative data from two independent experiments. Statistical significance was calculated using a paired two-tailed Student's *t*-test (**a-c**) or an unpaired two-tailed Student's *t*-test followed by Mann-Whitney test (**d-e**). Source data are provided as a Source Data file.

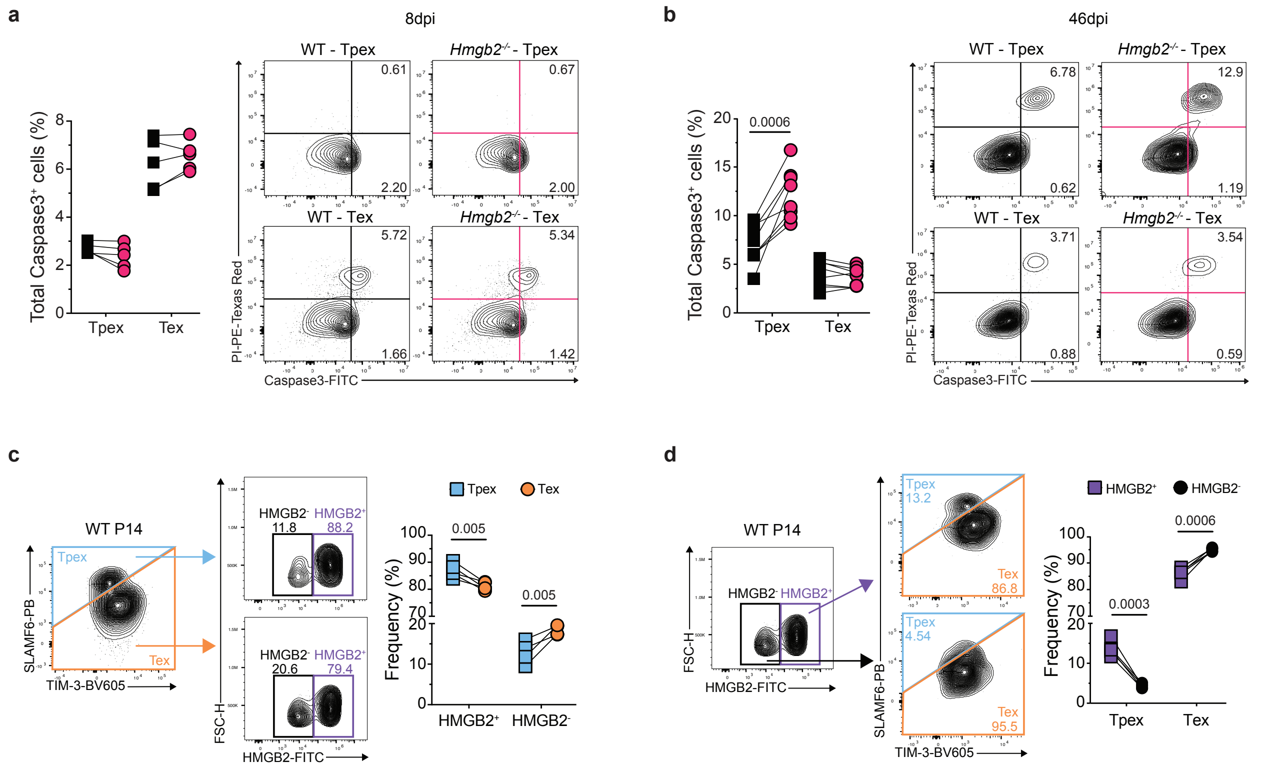

Supplementary Figure 8. HMGB2 regulation of Tpex cells during chronic LCMV infection Frequency of total Caspase3+ splenic WT and *Hmgb2-/-* progenitor exhausted (Tpex) and terminal exhausted (Tex) T cells at 8dpi (**a**) and 46dpi (**b**) Cl13; *n* = 5 (**a**), *n* = 8 (**b**). (**c**) Frequencies of HMGB2+ and HMGB2- cells within Tpex and Tex WT P14 T cells isolated from spleens at 8dpi Cl13; *n* = 5. (**d**) Frequencies of Tpex and Tex within HMGB2+ and HMGB2- WT P14 T cells isolated from spleens at 8dpi Cl13; *n* = 5. Data is mean ± s.e.m. Data are representative of two or more independent experiments. Statistical significance was calculated using a paired two-tailed Student's *t*-test. Source data are provided as a Source Data file.

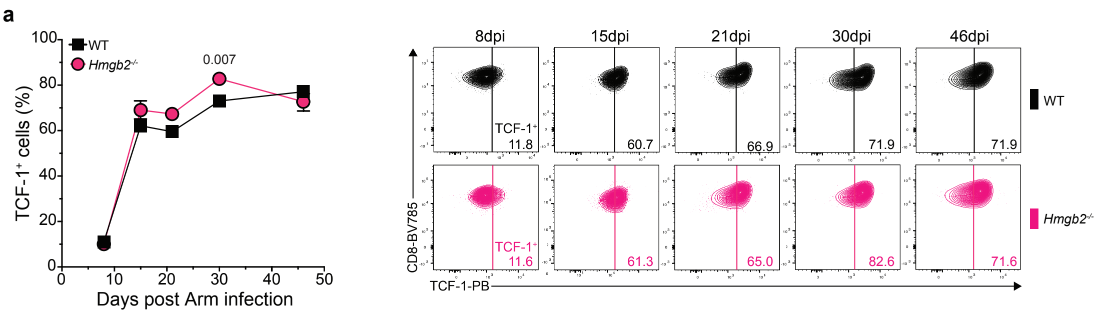

Supplementary Figure 9. TCF-1 expression in effector and memory *Hmgb2*$^{-/-}$ CD8^{+} T cells
(**a**) TCF-1 expression in co-transferred WT and *Hmgb2*$^{-/-}$ P14 T cells during LCMV Arm infection in the blood; *n* = 5. Data is mean ± s.e.m. Data are representative of three independent experiments. Statistical significance was calculated using a paired two-tailed Student's *t*-test. Source data are provided as a Source Data file.

Supplementary Table 1		
REAGENT OR RESOURCE	SOURCE	IDENTIFIER
Flow Cytometry Reagents		
anti-Bcl6 conjugated to APC (7D1)	BioLegend	Cat# 358506, RRID:AB_2562472
anti-CCR7 conjugated to BV605 (4B12)	BioLegend	Cat# 120125, RRID:AB_2715777
anti-CCR7 conjugated to PE (4B12)	BioLegend	Cat# 120106, RRID:AB_389358
anti-CD127 conjugated to BV605 (A7R34)	BioLegend	Cat# 135041, RRID:AB_2572047
anti-CD223 conjugated to PerCP5.5 (C9B7W)	BioLegend	Cat# 125212, RRID:AB_2561517
anti-CD279 conjugated to BV510 (29F.1A12)	BioLegend	Cat# 135241, RRID:AB_2715761
anti-CD279 conjugated to PE-Cy7 (29F.1A12)	BioLegend	Cat# 109110, RRID:AB_572017
anti-CD44 conjugated to APC-Cy7 (IM7)	BioLegend	Cat# 103028, RRID:AB_830785
anti-CD44 conjugated to PE (IM7)	BioLegend	Cat# 103008, RRID:AB_312959
anti-CD45.1 conjugated to APC (A20)	BioLegend	Cat# 110714, RRID:AB_313503
anti-CD45.1 conjugated to FITC (A20)	BioLegend	Cat# 110706, RRID:AB_313495
anti-CD45.1 conjugated to Pacific Blue (A20)	BioLegend	Cat# 110722, RRID:AB_492866
anti-CD45.1 conjugated to PE-Cy7 (A20)	BioLegend	Cat# 110730, RRID:AB_1134168
anti-CD45.2 conjugated to APC (104)	BioLegend	Cat# 109814, RRID:AB_389211
anti-CD45.2 conjugated to APC-Cy7 (104)	BioLegend	Cat# 109824, RRID:AB_830789
anti-CD45.2 conjugated to BV605 (104)	BioLegend	Cat# 109841, RRID:AB_2563485
anti-CD45.2 conjugated to FITC (104)	BioLegend	Cat# 109806, RRID:AB_313443
anti-CD45.2 conjugated to Pacific Blue (104)	BioLegend	Cat# 109820, RRID:AB_492872
anti-CD45.2 conjugated to PE (104)	BioLegend	Cat# 109808, RRID:AB_313445
anti-CD45.2 conjugated to PE-Cy7 (104)	BioLegend	Cat# 109830, RRID:AB_1186098
anti-CD62L conjugated to PE (MEL-14)	BioLegend	Cat# 104408, RRID:AB_313095
anti-CD62L conjugated to PerCP (MEL-14)	BioLegend	Cat# 104430, RRID:AB_2187124
anti-CD69 conjugated to PE-Cy7 (H1.2F3)	BioLegend	Cat# 104512, RRID:AB_493564

anti-CD8α conjugated to APC (53-6.7)	BioLegend	Cat# 100712, RRID:AB_312751
anti-CD8α conjugated to BV510 (53-6.7)	BioLegend	Cat# 100752, RRID:AB_2563057
anti-CD8α conjugated to BV605 (53-6.7)	BioLegend	Cat# 100744, RRID:AB_2562609
anti-CD8α conjugated to BV785 (53-6.7)	BioLegend	Cat# 100750, RRID:AB_2562610
anti-CD8α conjugated to Pacific Blue (53-6.7)	BioLegend	Cat# 100725, RRID:AB_493425
anti-CD8α conjugated to PE (53-6.7)	BioLegend	Cat# 100708, RRID:AB_312747
anti-CD8α conjugated to PE-Cy7 (53-6.7)	BioLegend	Cat# 100528, RRID:AB_312729
anti-CXCR5 conjugated to APC-Cy7 (L138D7)	BioLegend	Cat# 145526, RRID:AB_2566799
anti-HMGB2 unconjugated (ERP6302)	Abcam	Cat# ab133540
anti-IFN-γ conjugated to APC (XMG1.2)	BioLegend	Cat# 505810, RRID:AB_315404
anti-IFN-γ conjugated to FITC (XMG1.2)	BioLegend	Cat# 505806, RRID:AB_315400
anti-IL-2 conjugated to PE (JES6-5H4)	BioLegend	Cat# 503808, RRID:AB_315302
anti-KLRG1 conjugated to APC (2F1/KLRG1)	BioLegend	Cat# 138412, RRID:AB_10641560
anti-KLRG1 conjugated to FITC (2F1/KLRG1)	BioLegend	Cat# 138410, RRID:AB_10643582
anti-Ly108 conjugated to BV421 (13G3)	BD	Cat# 740090, RRID:AB_2739850
anti-Ly108 conjugated to PE (13G3)	BD	Cat# 561540, RRID:AB_561540
anti-TCF1/TCF7 conjugated to AF488 (C63D9)	Cell Signaling Technology	Cat# 6444S, RRID:AB_2797627
anti-TCF1/TCF7 conjugated to Pacific Blue (C63D9)	Cell Signaling Technology	Cat# 9066S, RRID:AB_2797696
anti-TCR Vß8.1.2 conjugated to FITC (MR5-2)	BD	Cat# 553185, RRID:AB_394694
anti-TCR Vα2 conjugated to PE (B20.1)	BioLegend	Cat# 127808, RRID:AB_1134183
anti-TNF-α conjugated to PE-Cy7 (MP6-XT22)	BioLegend	Cat# 506324, RRID:AB_2256076
anti-TOX conjugated to PE (REA473)	Miltenyi	Cat# 130-120-785, RRID:AB_2801785
Biotin anti-CD11b (M1/70)	BioLegend	Cat# 101204, RRID:AB_312787
Biotin anti-CD11c (N418)	BioLegend	Cat# 117304, RRID:AB_313773
Biotin anti-CD16/32 (93)	BioLegend	Cat# 101302, RRID:AB_312801

Biotin anti-CD19 (6D5)	BioLegend	Cat# 115504, RRID:AB_313639
Biotin anti-CD24 (M1/69)	BioLegend	Cat# 101804, RRID:AB_312837
Biotin anti-CD4 (GK1.5)	BioLegend	Cat# 100404, RRID:AB_312689
Biotin anti-CD45R/B220 (RA3-6B2)	BioLegend	Cat# 103204, RRID:AB_312989
Donkey anti-rabbit IgG conjugated to AF488 (Poly4064)	BioLegend	Cat# 406416, RRID:AB_2563203
Donkey anti-rabbit IgG conjugated to AF647 (poly4064)	BioLegend	Cat# 406414, RRID:AB_2563202
Granzyme B (GB12)	Fisher	Cat# 50-113-7520, RRID:AB_10372671
H-2D^b-GP$_{33-41}$ tetramer	NIH	Tetramer Core Facility Emory University
H-2D^b-GP$_{276-286}$ tetramer	NIH	Tetramer Core Facility Emory University
H-2D^b -NP$_{396-404}$ tetramer	NIH	Tetramer Core Facility Emory University
Ki-67 FITC (B56)	Fisher	Cat# BDB556026, RRID:AB_2266296
7-AAD	Fisher	Cat# BDB559925
CaspGLOW fluorescein active Caspase-3 kit	Life Technologies	Cat# 88-7004-42
eBioscience Foxp3/transcription factor fixation/permeabilization concentrate and diluent	Thermo Fisher Scientific	Cat# 00-5521-00
FITC BrdU Flow Kit	Fisher	Cat# BDB557891
Fixation/Permeabilization Solution Kit	BD Biosciences	Cat# 554714
Zombie Aqua Fixable Viability Kit	BioLegend	Cat# 423101
Western Blot		
anti-Histone H3	Abcam	Cat# ab1791, RRID: AB_302613
anti-Phospho-Histone H2A.X (Ser139) (20E3)	Cell Signaling Technology	Cat# 9718, RRID:AB_2118009
Chemicals, Peptides, and Recombinant Proteins		
Brefeldin A	Sigma	Cat# B7651-5MG
Dulbecco's modified Eagle's medium (DMEM)	Thermo Fisher Scientific	Cat# MT10016CV
Dulbecco's Phosphate-Buffered Salt Solution 1X	Thermo Fisher Scientific	Cat# MT21031CV
Fetal bovine serum (FBS)	Sigma	Cat# F0926-500ML
Geneticin Selective Antibiotic (G418 Sulfate)	Life Technologies	Cat# 10131027
HEPES Buffer	Thermo Fisher Scientific	Cat# MT25060CI
Ionomycin calcium salt	Sigma	Cat# I0634-1MG
MEM nonessential amino acid solution	Thermo Fisher Scientific	Cat# MT25025CI

Minimum essential medium eagle (MEM) without Glutamine	Thermo Fisher Scientific	Cat# MT15010CV
Penicillin-Streptomycin-L-Glutamine, 100X	Thermo Fisher Scientific	Cat# MT30009CI
Phorbol 12-myristate 13-acetate	Thermo Fisher Scientific	Cat# ICN19480401
Sodium Pyruvate 100mM solution	Thermo Fisher Scientific	Cat# MT25000CI
Trypsin-EDTA (0.25%), phenol red	Thermo Fisher Scientific	Cat# 25200072
Critical Commercial Assays		
Comet Assay Kit (15 tests)	Abcam	Cat# ab238544
EasyEights EasySep Magnet	Stemcell Technologies	Cat #18103
EasySep Mouse Streptavidin RapidSpheres Isolation Kit	Stemcell Technologies	Cat #19860
Lung Dissociation Kit, mouse	Miltenyi	Cat# 130-095-927
Experimental Models: LCMV		
LCMV Clone13 (Cl13)		Grew up in house
LCMV Armstrong (Arm)		Grew up in house
Experimental Models: Cell Lines		
Mouse: B16GP$_{33}$	Dr. Ananda Goldrath	Li et al., 2019
Experimental Models: Organisms/Strains		
C57BL/6 males	The Jackson Laboratory	Cat# 000664
Pepcb/BoyJ	The Jackson Laboratory	Cat# 002014
P14	Dr. Charles D. Surh (Scripps, SD)	
P14 *Hmgb2*$^{-/-}$	Dr. Marco Bianchi (San Raffaele, Milan)	
Software and Algorithms		
FlowJo 10.9.1	TreeStar	
Prism 9	GraphPad	
Rstudio 2023.06.1+524	Posit Software, PBC	
IPA	Qiagen	
Datasets		
D8 bulk RNA-sequencing on P14 cells (Arm and Cl13)	In this paper	GEO SuperSeries: GSE237813
D20 bulk RNA-sequencing on P14 cells (Cl13)	In this paper	GEO SuperSeries: GSE237813
D8 ATAC-sequencing on P14 cells (Cl13)	In this paper	GEO SuperSeries: GSE237813
