# Supplementary material for: HMGB2 regulates the differentiation and stemness of exhausted CD8+ T cells during chronic viral infection and cancer
Source: Nat Commun. 2023 Sep 13;14:5631. doi: 10.1038/s41467-023-41352-0 (PMC10499904; doi:10.1038/s41467-023-41352-0)
Supplement: Supplementary file 2 — Reporting Summary [file 41467_2023_41352_MOESM2_ESM.pdf]

nature portfolio

Corresponding author(s): Dr. Roberto Tinoco

Last updated by author(s): Jul 27, 2023

Reporting Summary

Nature Portfolio wishes to improve the reproducibility of the work that we publish. This form provides structure for consistency and transparency in reporting. For further information on Nature Portfolio policies, see our Editorial Policies and the Editorial Policy Checklist.

Statistics

For all statistical analyses, confirm that the following items are present in the figure legend, table legend, main text, or Methods section.

n/a	Confirmed	
☐	☒	The exact sample size (*n*) for each experimental group/condition, given as a discrete number and unit of measurement
☐	☒	A statement on whether measurements were taken from distinct samples or whether the same sample was measured repeatedly
☐	☒	The statistical test(s) used AND whether they are one- or two-sided<br>*Only common tests should be described solely by name; describe more complex techniques in the Methods section.*
☐	☒	A description of all covariates tested
☒	☐	A description of any assumptions or corrections, such as tests of normality and adjustment for multiple comparisons
☐	☒	A full description of the statistical parameters including central tendency (e.g. means) or other basic estimates (e.g. regression coefficient) AND variation (e.g. standard deviation) or associated estimates of uncertainty (e.g. confidence intervals)
☐	☒	For null hypothesis testing, the test statistic (e.g. *F*, *t*, *r*) with confidence intervals, effect sizes, degrees of freedom and *P* value noted<br>*Give P values as exact values whenever suitable.*
☒	☐	For Bayesian analysis, information on the choice of priors and Markov chain Monte Carlo settings
☒	☐	For hierarchical and complex designs, identification of the appropriate level for tests and full reporting of outcomes
☒	☐	Estimates of effect sizes (e.g. Cohen's *d*, Pearson's *r*), indicating how they were calculated

Our web collection on statistics for biologists contains articles on many of the points above.

Software and code

Policy information about availability of computer code

Data collection	Please see Supplementary Table 1 ("Datasets")
Data analysis	1. All flow cytometry data was analyzed using FlowJo v10.9.1 (Tree Star).<br>2. All statistical tests were run using Prism 9 (GraphPad Software)<br>3. For bulk RNA-sequencing: Post-processing of the run to generate FASTQ files was performed at the Institute for Genomics and Bioinformatics (UCI IGB). PcaHubert was used to identify any outlier samples, which were removed from further analysis67. The quality of the sequencing was first assessed using the fastQC tool (v0.11.9). Raw reads were then quality trimmed and filtered by a length of 20 bases using trimmomatic (v0.39). Trimmed reads were analyzed with the mouse Grcm38 reference genome using pseudo aligner Salmon (v1.2.1) and resulting quantification files were imported using R package tximport to get TPM values for all annotated mouse genes. Differential analysis was done using R package DESeq2 (v1.22.2) with an FDR cut off of 0.05. PCA was done using R packages DESeq2 and pheatmap.<br>4. For ATAC-sequencing: Paired ended reads from sequencing were QC analyzed with fastqQC (v.11.9) and aligned to mouse mm10 reference genome using bowtie2 (v2.4.1). Mitochondrial reads and reads mapped to dark list (ENCODE Stanford version) were excluded from the downstream analysis. Duplicated reads were removed using Picard tools (v2.27.1). A union peak list was created by merging processed reads from all samples and then calling peaks using MACS2 (v2.7.1) (-q 0.01 --keep-dup all -f BAMPE). The number of reads in each peak were then counted using featureCounts (Rsubread v2.6.4) to create a counts matrix. Normalization of counts matrix was performed using DESeq2 (v1.32.0). Differentially expressed peaks were determined using edgeR (v3.34.1) with an FDR cut-off of 0.05 and a |log10FC| cut-off of ≥ 0.3. Peaks were annotated using ChIPSeeker (v1.34.0).

For manuscripts utilizing custom algorithms or software that are central to the research but not yet described in published literature, software must be made available to editors and reviewers. We strongly encourage code deposition in a community repository (e.g. GitHub). See the Nature Portfolio guidelines for submitting code & software for further information.

Data

Policy information about availability of data

All manuscripts must include a data availability statement. This statement should provide the following information, where applicable:

- Accession codes, unique identifiers, or web links for publicly available datasets
- A description of any restrictions on data availability
- For clinical datasets or third party data, please ensure that the statement adheres to our policy

All data generated during this study are available within the paper. All sequencing data from this study will be deposited in the National Center for Biotechnology Information Gene Expression Omnibus (GEO) under GEO Series accession code XYZ. URL. Any other relevant data are available from the corresponding author on request.

Human research participants

Policy information about studies involving human research participants and Sex and Gender in Research.

Reporting on sex and gender	N/A
Population characteristics	N/A
Recruitment	N/A
Ethics oversight	N/A

Note that full information on the approval of the study protocol must also be provided in the manuscript.

Field-specific reporting

Please select the one below that is the best fit for your research. If you are not sure, read the appropriate sections before making your selection.

[x] Life sciences   [ ] Behavioural & social sciences   [ ] Ecological, evolutionary & environmental sciences

For a reference copy of the document with all sections, see nature.com/documents/nr-reporting-summary-flat.pdf

Life sciences study design

All studies must disclose on these points even when the disclosure is negative.

Sample size	No pre-experimental statistical methods, randomization, or blinding were used in animal experiments. Group sizes for experiments were selected based upon prior knowledge. Sample-size choice and assumption of normality were based on similar analyses in published studies; for adoptive transfer persistence experiments (e.g. PMID: 17420267) and for rechallenge experiments (e.g. PMID: 23644506).<br>For the end point of experiments, more than 5 mice per group were used. Based on previous studies (PMID: 35303066, PMID: 34326837, PMID: 27332735), these sample sizes allow for statistically valid comparisons.<br>For RNA- and ATAC-seq experiments, at each sample was collected from a pool of more than 5 mice following a standard set of previous publications from the field (e.g. PMID: 27939672, PMID: 33574619, PMID: 31207603).
Data exclusions	No data were excluded from the current study.
Replication	Results were confirmed in at least three independent experiments, unless otherwise indicated clearly in the figure legend.
Randomization	Age- and gender-matched animals were used for each experiment. Mice were allocated to groups randomly prior to experiments (simple randomization).
Blinding	Blinding was not performed due to requirements for cage labeling and data analysis was strictly quantitative and no objective.

Reporting for specific materials, systems and methods

We require information from authors about some types of materials, experimental systems and methods used in many studies. Here, indicate whether each material, system or method listed is relevant to your study. If you are not sure if a list item applies to your research, read the appropriate section before selecting a response.

Materials & experimental systems

n/a	Involved in the study	
☐	☒	Antibodies
☐	☒	Eukaryotic cell lines
☒	☐	Palaeontology and archaeology
☐	☒	Animals and other organisms
☒	☐	Clinical data
☒	☐	Dual use research of concern

Methods

n/a	Involved in the study	
☒	☐	ChIP-seq
☐	☒	Flow cytometry
☒	☐	MRI-based neuroimaging

Antibodies

Antibodies used

The following fluorochrome-conjugated antibodies were used (clone mentioned in parentheses):

From Abcam:
anti-HMGB2 unconjugated (ERP6302), Cat# ab133540, dilution 1:100

From BD:
anti-Ly108 conjugated to BV421 (13G3), Cat# 740090, dilution 1:200
anti-Ly108 conjugated to PE (13G3), Cat# 561540, dilution 1:200
anti-TCR Vß8.1.2 conjugated to FITC (MR5-2), Cat# 553185, dilution 1:200

From BioLegend:
anti-Bcl6 conjugated to APC (7D1), Cat# 358506, dilution 1:200
anti-CCR7 conjugated to BV605 (4B12), Cat# 120125, 1:200
anti-CCR7 conjugated to PE (4B12), Cat# 120106, dilution 1:200
anti-CD127 conjugated to BV605 (A7R34), Cat# 135041, dilution 1:200
anti-CD223 conjugated to PerCP5.5 (C9B7W), Cat# 125212, dilution 1:200
anti-CD279 conjugated to BV510 (29F.1A12), Cat# 135241, dilution 1:200
anti-CD279 conjugated to PE-Cy7 (29F.1A12), Cat# 109110, dilution 1:200
anti-CD44 conjugated to APC-Cy7 (IM7), Cat# 103028, dilution 1:200
anti-CD44 conjugated to PE (IM7), Cat# 103008, dilution 1:200
anti-CD45.1 conjugated to APC (A20). Cat# 110714, dilution 1:200
anti-CD45.1 conjugated to FITC (A20), Cat# 110706, dilution 1:200
anti-CD45.1 conjugated to Pacific Blue (A20), Cat# 110722, dilution 1:200
anti-CD45.1 conjugated to PE-Cy7 (A20), Cat# 110730, dilution 1:200
anti-CD45.2 conjugated to APC (104), Cat# 109814, dilution 1:200
anti-CD45.2 conjugated to APC-Cy7 (104), Cat# 109824, dilution 1:200
anti-CD45.2 conjugated to BV605 (104), Cat# 109841, dilution 1:200
anti-CD45.2 conjugated to FITC (104), Cat# 109806, dilution 1:200
anti-CD45.2 conjugated to Pacific Blue (104), Cat# 109820, dilution 1:200
anti-CD45.2 conjugated to PE (104), Cat# 109808, dilution 1:200
anti-CD45.2 conjugated to PE-Cy7 (104) , Cat# 109830, dilution 1:200
anti-CD62L conjugated to PE (MEL-14), Cat# 104408, dilution 1:200
anti-CD62L conjugated to PerCP (MEL-14), Cat# 104430, dilution 1:200
anti-CD69 conjugated to PE-Cy7 (H1.2F3), Cat# 104512, dilution 1:200
anti-CD8α conjugated to APC (53-6.7), Cat# 100712, dilution 1:200
anti-CD8α conjugated to BV510 (53-6.7), Cat# 100752, dilution 1:200
anti-CD8α conjugated to BV605 (53-6.7) , Cat# 100744, dilution 1:200
anti-CD8α conjugated to BV785 (53-6.7), Cat# 100750, dilution 1:200
anti-CD8α conjugated to Pacific Blue (53-6.7), Cat# 100725, dilution 1:200
anti-CD8α conjugated to PE (53-6.7), Cat# 100708, dilution 1:200
anti-CD8α conjugated to PE-Cy7 (53-6.7), Cat# 100528, dilution 1:200
anti-CXCR5 conjugated to APC-Cy7 (L138D7), Cat# 145526, dilution 1:200
anti-IFN-γ conjugated to APC (XMG1.2), Cat# 505810, dilution 1:100
anti-IFN-γ conjugated to FITC (XMG1.2), Cat# 505806, dilution 1:100
anti-IL-2 conjugated to PE (JES6-5H4), Cat# 503808, dilution 1:100
anti-KLRG1 conjugated to APC (2F1/KLRG1), Cat# 138412, dilution 1:200
anti-KLRG1 conjugated to FITC (2F1/KLRG1), Cat# 138410, dilution 1:200
anti-TCR Vα2 conjugated to PE (B20.1), Cat# 127808, dilution 1:200
anti-TNF-α conjugated to PE-Cy7 (MP6-XT22), Cat# 506324, dilution 1:100
Biotin anti-CD11b (M1/70), Cat# 101204, dilution 1:30
Biotin anti-CD11c (N418), Cat# 117304, dilution 1:30
Biotin anti-CD16/32 (93), Cat# 101302, dilution 1:30
Biotin anti-CD19 (6D5), Cat# 115504, dilution 1:30
Biotin anti-CD24 (M1/69), Cat# 101804, dilution 1:30

	Biotin anti-CD4 (GK1.5), Cat# 100404, dilution 1:30<br>Biotin anti-CD45R/B220 (RA3-6B2), Cat# 103204, dilution 1:30<br>Donkey anti-rabbit IgG conjugated to AF488 (Poly4064), Cat# 406416, dilution 1:200<br>Donkey anti-rabbit IgG conjugated to AF647 (poly4064), Cat# 406414, dilution 1:200<br><br>From Cell Signaling:<br>anti-TCF1/TCF7 conjugated to AF488 (C63D9), Cat# 6444S, dilution 1:100<br>anti-TCF1/TCF7 conjugated to Pacific Blue (C63D9), Cat# 9066S, dilution 1:100<br><br>From Fisher:<br>Granzyme B (GB12), Cat# 50-113-7520, dilution 1uL/well<br>Ki-67 FITC (B56), Cat# BDB556026, dilution 1:20<br>7-AAD , Cat# BDB559925, dilution 1:20<br><br>From Miltenyi:<br>anti-TOX conjugated to PE (REA473), Cat# 130-120-785, dilution 1:100<br><br>From National Institute of Health (NIH) tetramer core:<br>H-2Db-GP33-41 tetramer, dilution 1:200<br>H-2Db-GP276-286 tetramer, dilution 1:200<br>H-2Db -NP396-404 tetramer, dilution 1:200
Validation	All antibodies used in this study are commercially available. Validation of individual antibodies has been performed by respective manufacturers and validation data are available on the manufacturer's respective websites. All flow panels were validated before use.

Eukaryotic cell lines

Policy information about cell lines and Sex and Gender in Research

Cell line source(s)	Mouse B16GP33 melanoma cells were obtained from Dr. Ananda Goldrath (UCSD).
Authentication	Cell lines were passaged two times per week and underwent a minimum of four passages before injections. To mitigate murine cell line adaptations while in culture, cells were cultured for a maximum of two months in vitro. Early passages were frozen down for future use. No additional cell authentication was performed
Mycoplasma contamination	All cell lines were free of mycoplasma.
Commonly misidentified lines<br>(See ICLAC register)	B16GP33 cells are not listed in the ICLAC list of misidentified cell lines.

Animals and other research organisms

Policy information about studies involving animals; ARRIVE guidelines recommended for reporting animal research, and Sex and Gender in Research

Laboratory animals	C57BL/6 male mice (CD45.2+) from The Jackson Laboratory were used at 6-8 weeks of age for the majority of experiments. Alternatively, Pepcb/BoyJ male mice (CD45.1+) were used for some single transfer experiments. P14 mice transgenic for a TCR recognizing the H-2Db GP33-41 epitope of LCMV were originally obtained from Dr. Charles D. Surh (Scripps) and then bred at University of California, Irvine (UCI). P14 Hmgb2-/- mice were obtained from Dr. Marco Bianchi (San Raffaele). Mice used in experiments were age- and sex-matched. Mice were maintained in a specific pathogen-free animal facility at UCI.
Wild animals	Study did not involve wild animals.
Reporting on sex	Findings did not apply to one sex. Sex of P14 and P14 Hmgb2-/- donor mice did not influence results.
Field-collected samples	Study did not involve field-collected samples.
Ethics oversight	All experimental animal procedures were approved by the Institutional Animal Care and Use Committee of University of California, Irvine (AUP-21-124) and complied with all relevant ethical regulations for animal testing and research.

Note that full information on the approval of the study protocol must also be provided in the manuscript.

Flow Cytometry

Plots

Confirm that:

- [x] The axis labels state the marker and fluorochrome used (e.g. CD4-FITC).
- [x] The axis scales are clearly visible. Include numbers along axes only for bottom left plot of group (a 'group' is an analysis of identical markers).
- [x] All plots are contour plots with outliers or pseudocolor plots.
- [x] A numerical value for number of cells or percentage (with statistics) is provided.

Methodology

Sample preparation	Single-cell suspensions were generated by mechanically homogenizing spleens and lymph nodes using a 70µm cell strainer. Red blood cells were lysed using ACK lysis buffer (ThermoFisher). When necessary, cells were sorted with a FACSAria (BD) to >95% purity. Before sorting and adoptive transfers, CD8+ T cells were enriched from total splenocytes using negative selection with the following biotinylated antibodies: CD4 (GK1.5), B220 (RA3-6B2), CD19 (6D5), CD24 (M1/69), CD11b (M1/70), and CD11c (N418). Non-CD8+ cells were removed by mixing labeled cell suspension with Streptavidin RapidSpheres (Stemcell technologies) and incubation on an EasyEights™ EasySep™ Magnet (Stemcell technologies).
Instrument	All data were collected on a Novocyte3000 (Agilent)
Software	All data were analyzed using FlowJo Software v10.9 (Tree Star)
Cell population abundance	Purity of post-sort samples was ≥95% as assessed on flow cytometer.
Gating strategy	FSC-H/SSC-H was used to gate on lymphocytes. Then doublets were excluded through FSC-H/FSC-A and SSC-H/SSC-A. CD8+ T cells were gated as CD8α positive. Donor cells were gated on their distinct congenic marker(s) using CD45.1/CD45.2 gating. A representative general gating strategy is also depicted in Supplemental Figure 1a.

- [x] Tick this box to confirm that a figure exemplifying the gating strategy is provided in the Supplementary Information.
